# The up-regulation of SYNCRIP promotes the proliferation and tumorigenesis via DNMT3A/p16 in colorectal cancer

**DOI:** 10.1038/s41598-024-59575-6

**Published:** 2024-09-16

**Authors:** Chenglong Li, Tailiang Lu, Hongxi Chen, Zhige Yu, Chaowu Chen

**Affiliations:** Department of Gastrointestinal Surgery, Hunan Provincial People’s Hospital (The First Affiliated Hospital of Hunan Normal University), Changsha, 410005 Hunan Province China

**Keywords:** Colorectal cancer, hnRNPs, DNA methyltransferases, Tumor suppressor, Tumor growth, Oncogenes, Colon cancer

## Abstract

Heterogeneous nuclear ribonucleoproteins (hnRNPs), a group of proteins that control gene expression, have been implicated in many post-transcriptional processes. SYNCRIP (also known as hnRNP Q), a subtype of hnRNPs, has been reported to be involved in mRNA splicing and translation. In addition, the deregulation of SYNCRIP was found in colorectal cancer (CRC). However, the role of SYNCRIP in regulating CRC growth remains largely unknown. Here, we found that SYNCRIP was highly expressed in colorectal cancer by analyzing TCGA and GEPIA database. Furthermore, we confirmed the expression of SYNCRIP expression in CRC tumor and CRC cell lines. Functionally, SYNCRIP depletion using shRNA in CRC cell lines (SW480 and HCT 116) resulted in increased caspase3/7 activity and decreased cell proliferation, as well as migration. Meanwhile, overexpression of SYNCRIP showed opposite results. Mechanistically, SYNCRIP regulated the expression of DNA methyltransferases (DNMT) 3A, but not DNMT1 or DNMT3B, which affected the expression of tumor suppressor, p16. More importantly, our in vivo experiments showed that SYNCRIP depletion significantly inhibited colorectal tumor growth. Taken all together, our results suggest SYNCRIP as a potent therapeutic target in colorectal cancer.

## Introduction

Colorectal cancer (CRC) is one of the most frequent malignant tumors worldwide and is the second leading cause of cancer-related deaths^1^. CRC is a multifactorial disease, and the risk factors include environmental, genetic, and biochemical factors, such as smoking, alcohol, age, and obesity^2,3^. The identification of diagnostic biomarkers and targeted therapy remain the major challenge of CRC treatment. Recently, significant progress has been made in the targeted therapy of CRC; the drugs targeting EGFR, PD-1/PD-L1, BRAF, CTLA-4, and NTRK have been approved to treat CRC patients and achieved great benefits^4,5^. However, despite the advances in therapeutic plans, the five-year survival of CRC patients remains low. Thus, it is urgent to understand the fundamental mechanism of tumorigenesis and identify novel effective therapeutic targets.

Heterogeneous nuclear ribonucleoproteins (hnRNPs) represent a large group of RNA-binding proteins that regulate multiple posttranscriptional events, such as alternative splicing, mRNA stabilization, RNA degradation and translational regulation^6^. The hnRNPs family members share general features and similar protein structure, consisting of at least one RNA-binding domain combines with an RGG box or acidic domain for protein–protein interaction or RNA binding^7^. Considering their key function in the cellular nucleic acid metabolism, the role of hnRNPs in regulating gene expression has gained more and more interest in disease research. Aberrant hnRNPs expression can cause a wide variety of diseases, including cancer. In lung cancer, hnRNP A1 knockdown inhibited lung adenocarcinoma cell proliferation through cell cycle arrest at G0/G1 phase^8^. In addition, hnRNP K promoted the progression of lung cancer by inhibiting the p53‐dependent signaling pathway^9^. In colorectal cancer, hnRNPA1 is augmented and stabilizeoncogenic mRNAs, such as cyclin D1 and c-Myc^10,11^. Also, the expression of hnRNP K is correlated with the prognosis of CRC^12^. HnRNP G**,** which is specifically expressed in the testis, expresses in colorectal cancer cells, and accelerates cell growth mediating ZDHHC11 mRNA stabilization^13^. More importantly, a study constructed siRNAs targeting 20 representative hnRNPs and discovered that all these hnRNPs promoted the progression of CRC^14^. hnRNP Q (also called SYNCRIP) is an AU-rich RNA-binding protein and have multifunction in regulating mRNAs, including mRNA splicing, mRNA editing, transport, turnover, and translation regulation^15–17^. Two studies showed that one of the hnRNP Q isoforms, hnRNP Q1, contributes to cell proliferation and tumorigenesis in colorectal cancer^18,19^. However, the mechanism remains largely unknown.

DNA methylation is one of the predominant epigenetic modifications in mammals and is controlled by DNA methyltransferases (DNMTs)^20^. In mammals, three catalytically active DNMTs (DNMT1, DNMT3A and DNMT3B) have been identified^21^. Elevated levels of DNMT1, DNMT3A and DNMT3B have been reported in various tumors, including hepatic, prostate, colorectal, and breast cancers^22–25^. Recently, several studies have shown that dysregulation of DNMT3A leads to oncogenesis, including colorectal cancer^26,27^. However, the regulatory mechanism of DNMT3A expression in CRC has not been well-defined and needs further investigation. In the present study, we confirmed the function of SYNCRIP in regulating colorectal cancer cells growth in vitro and in vivo and proved that the expression of DNMT3A was regulated by SYNCRIP, which suggested SYNCRIP as a potential therapeutic target for colorectal cancer.

## Materials and methods

### Antibodies

Antibodies against SYNCRIP (#PA5-50986) were obtained from Thermo Fisher Scientific (Waltham, MA). Antibodies against DNMT1 (#5032) and GAPDH (#5174) were obtained from Cell Signaling (MA, USA); Antibodies against DNMT3A (#20954-1-AP), DNMT3B (#26971-1-AP), and p16 (#10883-1-AP) were obtained from Proteintech (IL, USA).

### Cell culture

Human colorectal cancer cell lines SW480, SW620, HT29, HCT8, and HCT116 were purchased from the American Type Culture Collection (ATCC, Manassas, VA, USA) and cultured in DMEM/F12 medium (Lonza, Basel, Switzerland) supplemented with 10% (v/v) FBS (Gibco, Waltham, MA). Human colon mucosal epithelial cell line NCM460 were purchased from INCELL (TX, USA) and cultured in DMEM (Gibco, Waltham, MA) supplemented with 10% (v/v) FBS (Gibco, Waltham, MA) and penicillin/streptomycin.

### Short hairpin RNA (shRNA) knockdown

SYNCRIP shRNAs and DNMT3A were purchased from Sigma-Aldrich (MO, USA). The shRNA sequences were as follow: SYNCRIP shRNA1: 5′-GCAAAGTAACAGAGGGTCTTA-3′; SYNCRIP shRNA2: 5′-GTATGACGATTACTACTATTA-3′; DNMT3A shRNA1: 5′-CCACCAGAAGAAGAGAAGAAT-3′; shRNA plasmids were co-transfected with packaging constructs according to the manufacture’s instruction to package the lentiviruses. The virus particles were collected 48 h after transfection. SW480 and HCT116 cells were infected with lentivirus mixed with 8 μg/ml polybrene. Stable clones were established by puromycin (1 mg/mL) selection.

### Plasmid transfection

DNMT3A overexpression plasmid was purchased from Origene (#RC208192, MD USA). Briefly, SW480 and HCT116 cells were plated onto 6 well plate, and grew to 80% ~ 90% confluency. 2 μg empty vector or DNMT3A plasmid were transfected using Lipofectamine 2000 (Invitrogen, MA, USA) according to the manufacturer’s instructions. The cells were harvested after transfection for 24 h.

### Caspase-3/7 activity assay

Caspase-3/7 activity was measured using Apo-ONE™ Homogeneous Caspase-3/7 Assay (Promega Corporation, Madison, WI, USA) according to the protocol provided by the manufacturer.

### Cell proliferation and survival assay

Cell proliferation was assayed using Cell Counting Kit-8 assay (CCK8) kit following the manufacture’s instruction (Jiangsu KeyGENBioTECH Corp., Ltd, China). 3000 SW480 and HCT116 cells were seeded in 96-well plate after infected with SYNCRIP shRNA or transfected with SYNCRIP plasmid. At the indicated time, 10 μl CCK8 reagents were added into each well and incubated at 37 ◦C for 2 h, and then the absorbance at 450 nm was recorded.

Cell survival was assessed using trypan blue staining. The dead cells were blue stained and counted manually using hemocytometer.

### Migration assay

Migration assay was conducted using 8 μm transwell inserts (Corning, NY, USA). Briefly, 1 × 10^5^ SW480 and HCT116 cells infected with SYNCRIP shRNA or transfected with SYNCRIP plasmid in 100 μl serum free medium was added into the inserts and then placed in 24-well plate that fulfilled with 600 μl complete growth medium. The inserts were washed with PBS, fixed with 4% formaldehyde, and stained with 0.5% crystal violet after 24 h incubation at 37 °C. Five randomly fields were acquired using microscope.

### Wound healing assay

5 × 10^5^ SW480 cells infected with SYNCRIP shRNA or transfected with SYNCRIP plasmid were seeded 6-well plate; the cells were scratched with a 1 ml pipette tip after 100% confluent. The images were then captured using a microscope equipped with a digital camera. The images were recorded again after 24 h incubation at 37 °C.

### RNA isolation and quantitative reverse‐transcription PCR (qRT‐PCR)

Total RNA was isolated with TRIzol (Vazyme, Nanjing, China) following the manufacturer's instructions and the cDNA was synthesized using script cDNA synthesis kit (Bio-Rad, CA, USA). Quantitative PCR was performed using SYBR Premix Ex TaqII (TaKaRa, Japan) on CFX96 real-time PCR detection system (Bio-Rad, USA) with the following primers: SYNCRIP, forward: 5′-TGAGTGGTAAAGTCAAGGTCTGG-3′, reverse: 5′-GGCAAGGTTGCGTACAAACAGC-3′; DNMT1, forward: 5′-AGGTGGAGAGTTATGACGAGGC-3′, reverse: 5′-GGTAGAATGCCTGATGGTCTGC-3′; DNMT3A, forward: 5′-CCTCTTCGTTGGAGGAATGTGC-3′, reverse: 5′-GTTTCCGCACATGAGCACCTCA-3′; DNMT3B, forward: 5′-TAACAACGGCAAAGACCGAGGG-3′, reverse: 5′-TCCTGCCACAAGACAAACAGCC-3′; p16, forward: 5′-CTCGTGCTGATGCTACTGAGGA-3′, reverse: 5′-GGTCGGCGCAGTTGGGCTCC-3′; GAPDH, forward: 5′-AATCCCATCACCATCTTCCAG-3′, reverse: 5′-AAATGAGCCCCAGCCTTC-3′. Values for each gene were normalized to the expression of GAPDH.

### Western blot analysis

Total protein was extracted from SW480 and HCT116 cells using RIPA buffer (Invitrogen, MA, USA) supplemented with protease and phosphatase inhibitor cocktail (Sigma-Aldrich, MO, USA) and quantified using the BCA detecting kit according to the manufacturer's instructions. A total of 20 μg proteins were subjected to 10% SDS-PAGE gel separation, transferred to a nitrocellulose membrane, and blocked with 5% milk for 1 h at room temperature. The membranes were cut based on the molecular weight before incubation with the primary antibodies at 4 °C overnight. After three times wash with PBST, the membrane was incubated with secondary antibodies for 1 h at room temperature. Protein bands were visualized using Super Signal West Pico Chemiluminescent Substrate (Thermo Scientific, MA, USA), and the images were captured with the ChemiDoc Imaging system (Bio-Rad, CA, USA). The densitometric quantification was performed using Image J software.

### In-vivo xenograft experiment

5.0 × 10^6^ SW480 cells infected with scr or SYNCRIP shRNA in 50 µl PBS were mixed with equal volume of matrigel. The mixture was then subcutaneously injected in the flank of six-weeks-old male NOD/SCID (*The Jackson Laboratory,* Stock No: 001303) mice. The tumor size was measured at the indicated times after injection. Tumor size was calculated by 0.5 × (long diameter) × (short diameter)^2^. The mice were euthanized by cervical dislocation and tumor weight was measured after 4 weeks. All animal experiments were conducted according to the NIH Guide for the Care and Use of Laboratory Animals.

All procedures agreed with the Guide for the Care and Use of Laboratory Animals of the National Institutes of Health and approval was obtained by the Ethics Committee of the Hunan Normal University. All procedures performed in our research followed the recommendations in the ARRIVE guidelines.

### Database data

The SYNCRIP genetic alterations in colorectal cancer patients were obtained by searching SYNCRIP in cBioPortal database (www.cbioportal.org)^28^. The expression of SYNCRIP in colorectal tumor and normal tissue, different tumor types, and different disease stages was obtained by searching SYNCRIP in GEPIA (Gene Expression Profiling Interactive Analysis) database (http://gepia.cancer-pku.cn)^29^. The correlations analysis between SYNCRIP and DNMT3A, SYNCRIP and p16 were performed using Pearson correlation based on TCGA Tumor in GEPIA database.

### Statistical analysis

Data were presented as mean ± SD from three independent experiments. *P* value was determined using paired Student’s t-test, and a P value ˂ 0.05 was considered statistically significant. SPSS version 20.0 (SPSS, Inc) was used to perform the statistical analysis.

## Results

### SYNCRIP was up-regulated in human colorectal cancer

**Figure 1:**
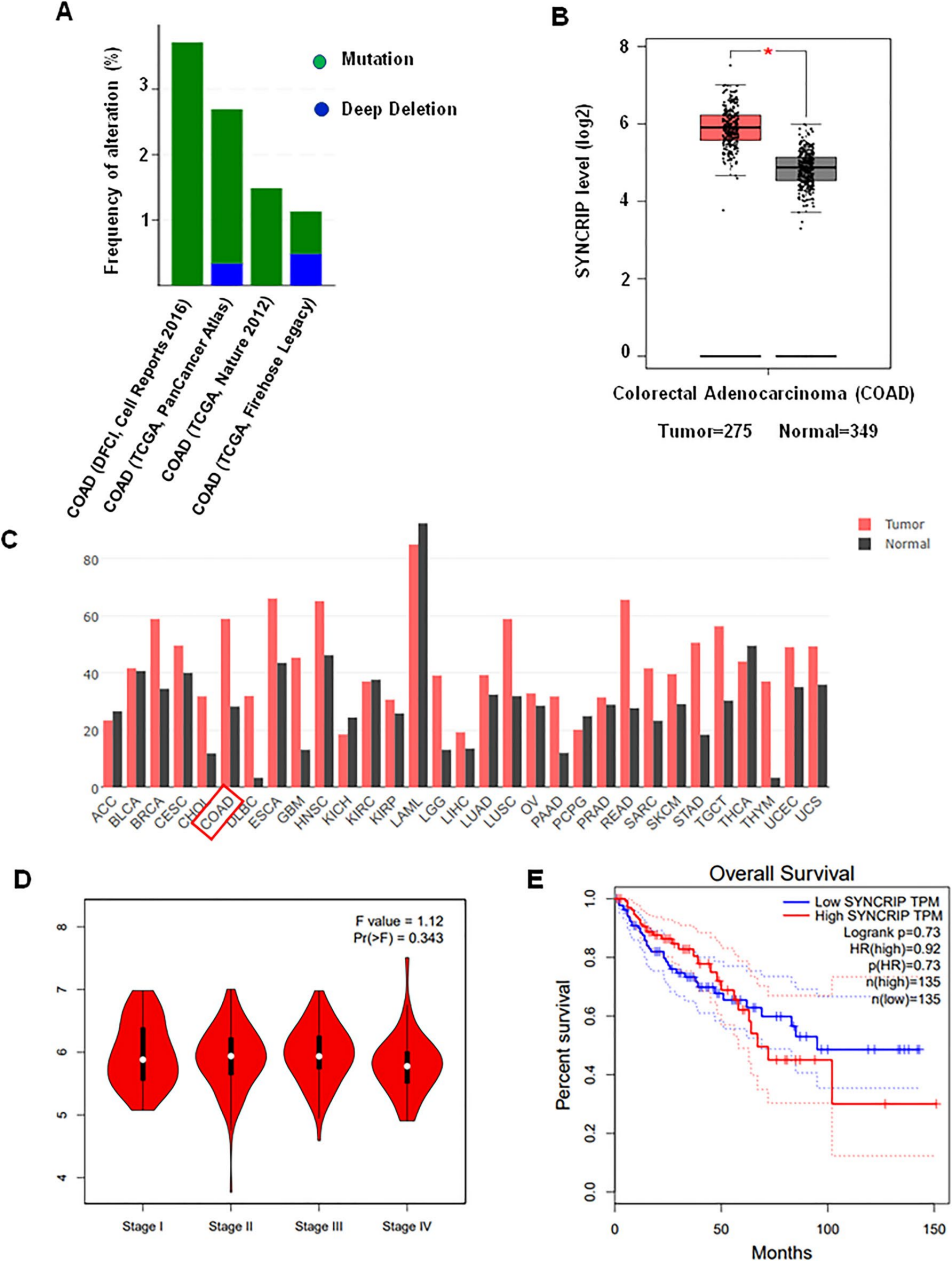
Database analysis of SYNCRIP expression in colorectal cancer. (**A**) TCGA database showed gene alteration frequency of SYNCRIP in colorectal cancer. (**B**, **C**) Expression level of SYNCRIP in tumor is higher than in normal tissue, shown by GEPIA database. (**D**) Expression level of SYNCRIP in different stage of colorectal cancer. (**E**) The overall survival rate of patients with high or low level of SYNCRIP.

To explore the potential function of SYNCRIP in colorectal cancer, we first analyzed the expression of SYNCRIP using publicly available database TCGA (The Cancer Genome Atlas) and GEPIA (Gene Expression Profiling Interactive Analysis). In TCGA database, the ratio of SYNCRIP genetic alterations in colorectal cancer patients was around 3% (Fig. 1A). More importantly, the expression level of SYNCRIP in colorectal tumor (n = 275) is significantly higher than in normal tissue (n = 349) (Fig. 1B,C. However, no significant difference of SYNCRIP expression was observed between different stages (Stage I-Stage IV) (Fig. 1D), which suggested that SYNCRIP is not one of the stage-specific differentially expressed genes. Furthermore, the CRC patients with higher SYNCRIP expression showed lower overall survival rate (Fig. 1E).

**Figure 2:**
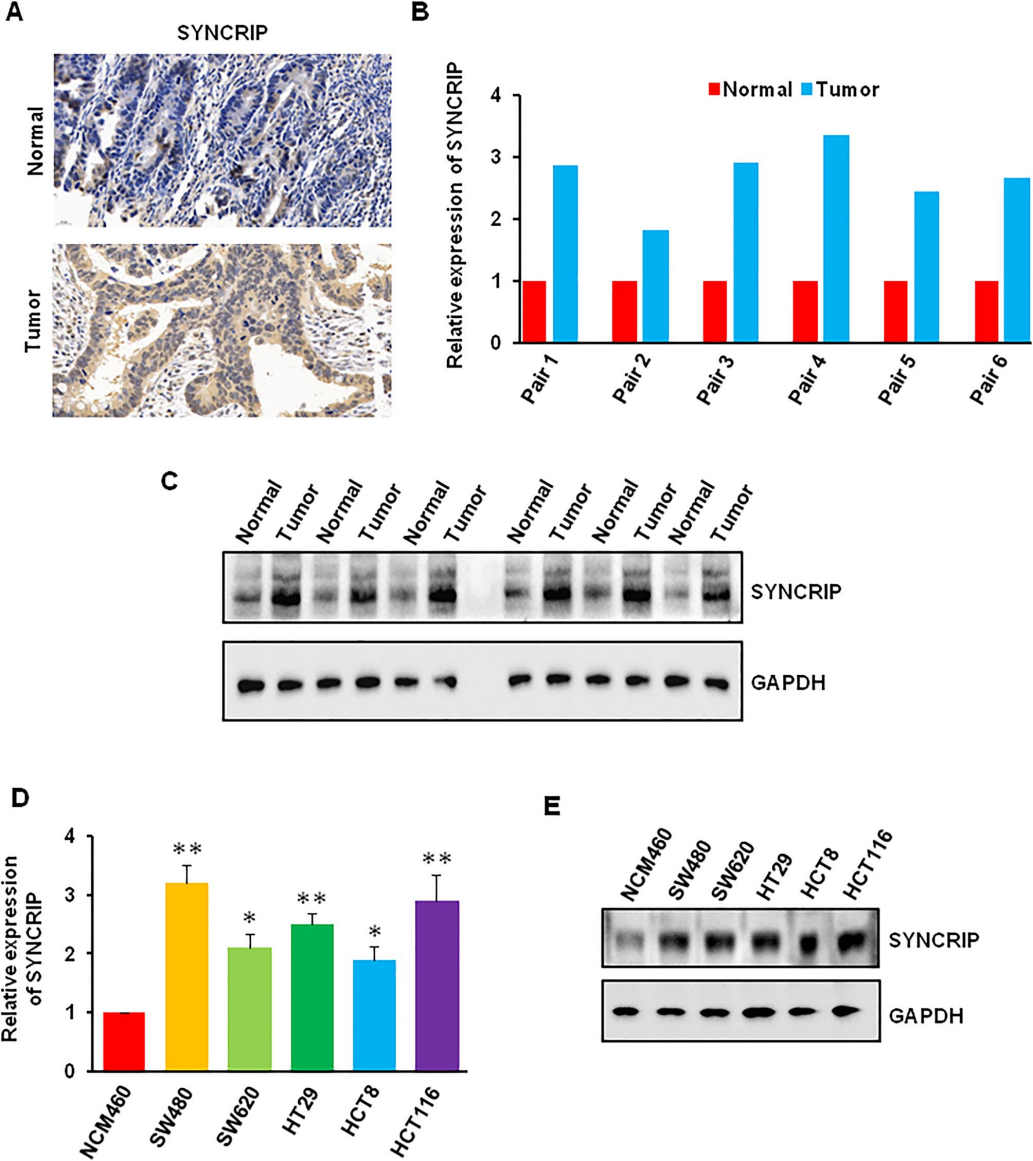
The expression of SYNCRIP in colorectal cancer cells. (**A**) Immunohistochemistry staining of SYNCRIP in CRC tumor tissue and adjacent normal tissues. (**B**, **C**) The mRNA and protein level of SYNCRIP in human colorectal cancer tissues and adjacent normal tissues was detected by QRT-PCR (**B**) and Western blotting (**C**). (**D**, **E**) The mRNA and protein level of SYNCRIP in human normal colon mucosal epithelial cell and colorectal cancer cell lines was detected by QRT-PCR (**D**) and Western blotting (**E**). *P ˂ 0.05, **P ˂ 0.01.

To further confirm the expression of SYNCRIP in CRC, we detected the mRNA and protein level of SYNCRIP in tumor tissue and adjacent normal tissues. Our immunohistochemistry staining showed that the positive rate of SYNCRIP was higher in CRC tissues than in normal tissues (Fig. 2A). In addition, we found that the mRNA and protein level of SYNCRIP was significantly higher in tumor tissue (Figs. 2B,C, S1A). We also compared the expression of SYNCRIP in normal human colon mucosal epithelial cell line (NCM460) and CRC cell lines (SW480, SW620, HT29, HCT8, and HCT116). Consistent with results from patients’ sample, SYNCRIP was highly expressed in CRC cell lines compared with normal NCM460 cell line (Figs. 2D,E, S1B).

### SYNCRIP regulated colorectal cancer cells apoptosis, proliferation, and motility

**Figure 3:**
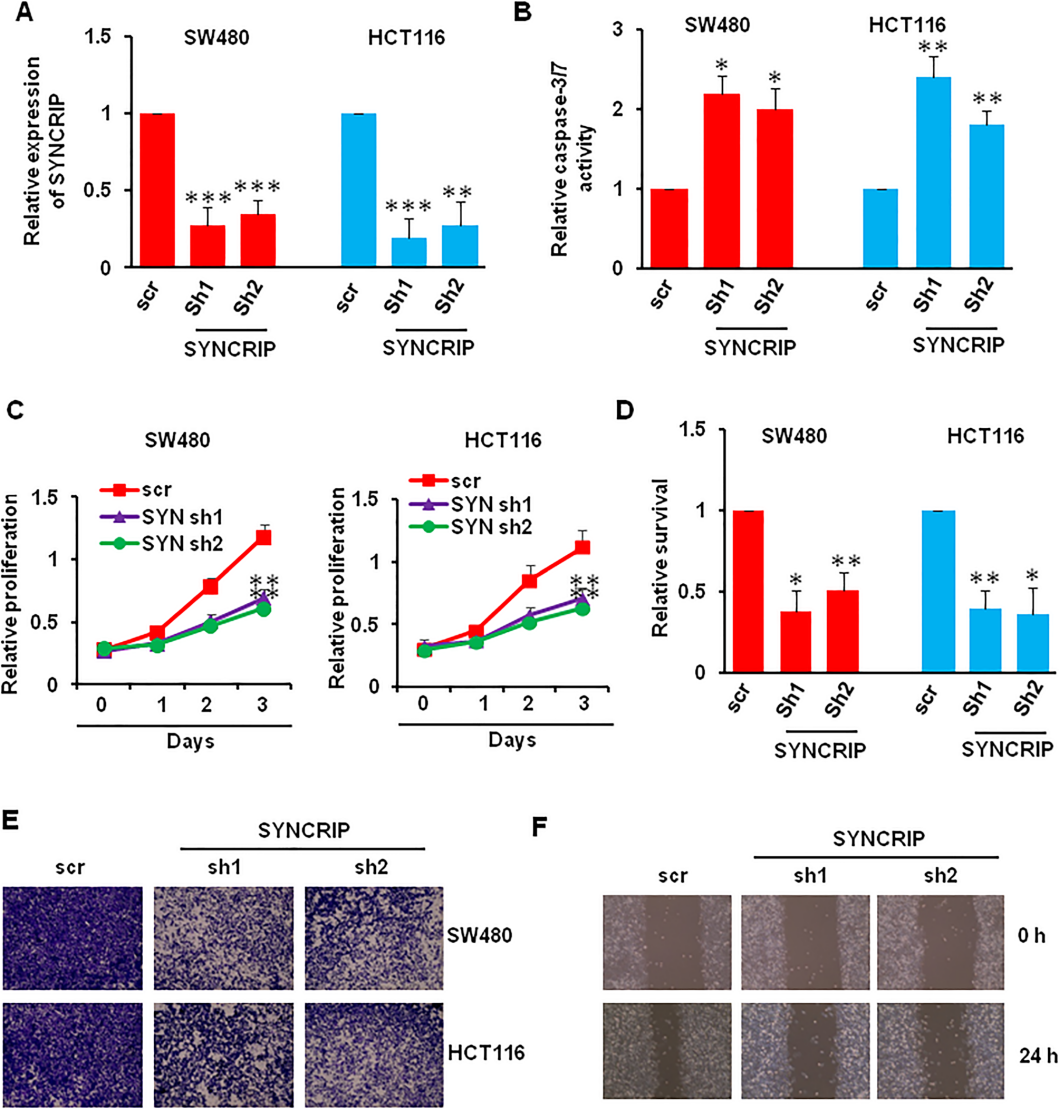
Effect of SYNCRIP depletion on cells apoptosis, proliferation, and migration. (**A**) The knockdown efficiency of SYNCRIP in colorectal cancer cells were detected by QRT-PCR. **P ˂ 0.01, ***P ˂ 0.001. (**B**) The activity of caspase3/7 was measured after SYNCRIP knockdown. *P ˂ 0.05, **P ˂ 0.01. (**C**, **D**) The proliferation and survival of SW480 and HCT116 cells after SYNCRIP depletion were measured using CCK-8 assay (**C**) and trypan blue stain (**D**). *P ˂ 0.05, **P ˂ 0.01. (**E**) Transwell migration assay of SW480 and HCT116 cells after SYNCRIP knockdown. (**F**) Wound healing assay of SW480 cells after SYNCRIP knockdown.

Our previous results showed highly expressed SYNCRIP in colorectal cancer cell, which suggested that SYNCRIP could play important role in CRC. To investigate the role of SYNCRIP in CRC, we knockdown SYNCRIP in SW480 and HCT116 cells using shRNA. Here, we tested two different shRNAs, and about 80% SYNCRIP was knockdown using both shRNAs in SW480 and HCT116 cells (Fig. 3A). Interestingly, SYNCRIP depletion increased the activity of caspase-3/7, which indicated that the apoptosis was increased (Fig. 3B). Furthermore, the cell proliferation and survival were also impaired after SYNCRIP depletion (Fig. 3C,D). To investigate the function of SYNCRIP on cell motility, we performed trans-well migration and wound healing assays. The motility of SW480 and HCT116 cells were inhibited in SYNCRIP shRNA infected cells compared with scramble (scr) infected cells (Figs. 3E,F, S2).

**Figure 4:**
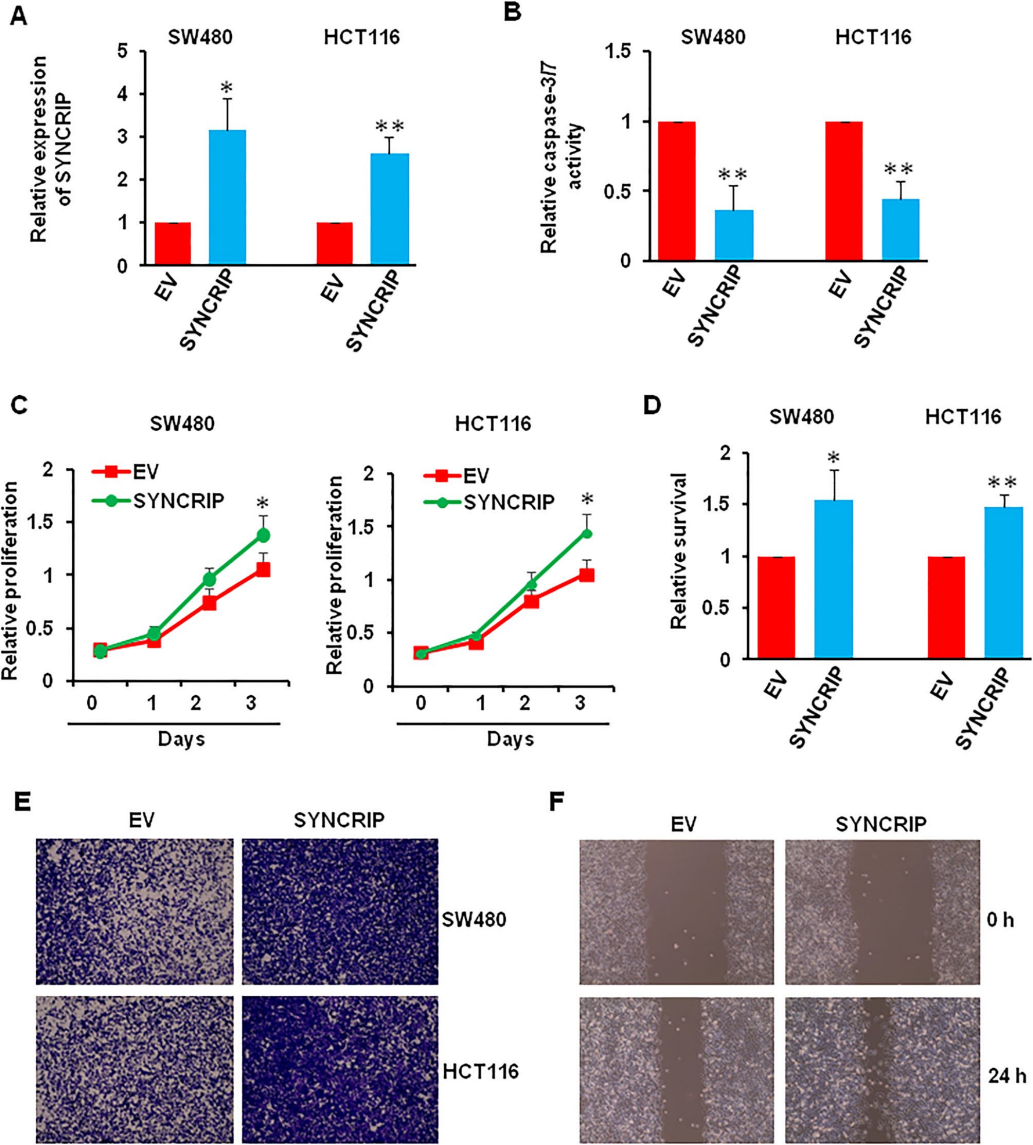
Effect of SYNCRIP overexpression on cells apoptosis, proliferation, and migration. (**A**) The overexpression efficiency of SYNCRIP in colorectal cancer cells were detected by QRT-PCR. *P ˂ 0.05, **P ˂ 0.01. (**B**) The activity of caspase3/7 was measured after SYNCRIP overexpression. **P ˂ 0.01. (**C**, **D**) The proliferation and survival of SW480 and HCT116 cells after SYNCRIP overexpression were measured using CCK- 8 assay (**C**) and trypan blue stain (**D**). *P ˂ 0.05, **P ˂ 0.01. (**E**) Transwell migration assay of SW480 and HCT116 cells after SYNCRIP overexpression. (**F**) Wound healing assay of SW480 cells after SYNCRIP overexpression.

Next, we wonder whether SYNCRIP overexpression has the reverse effects on cell apoptosis, proliferation, and motility. SYNCRIP plasmid and empty vector were used to transfect SW480 and HCT116 cells, about 2.5 times level of SYNCRIP was observed after SYNCRIP plasmid transfection (Fig. 4A). Not surprisingly, the activity of caspase-3/7 decreased, while the proliferation and motility increased after SYNCRIP overexpression, which indicated that SYNCRIP overexpression inhibited CRC cell apoptosis, but promoted cell proliferation and migration (Figs. 4B–F, S3).

### SYNCRIP regulated colorectal cancer cell proliferation and motility via regulating DNMT3A expression

**Figure 5:**
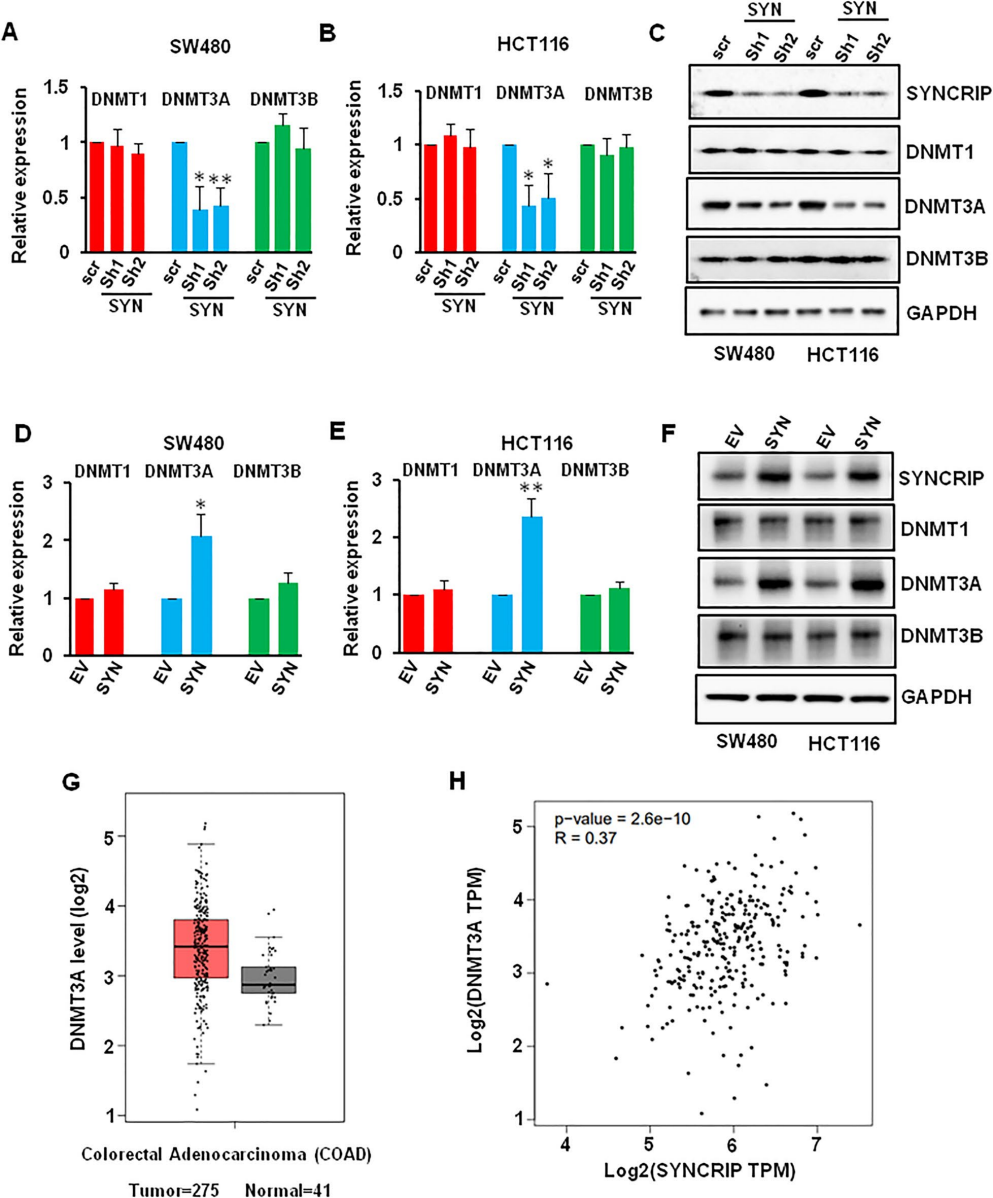
Effect of SYNCRIP on regulating the expression of DNMT3A. (**A**–**C**) SW480 and HCT116 cells were infected with scr or SYNCRIP shRNA (SYN sh) for 72 h, the mRNA and protein level of DNMTs were detected by QRT-PCR (**A**, **B**) and Western blotting (**C**). *P ˂ 0.05, **P ˂ 0.01. (**D**–**F**) SW480 and HCT116 cells were transfected with empty vector (EV) and SYNCRIP plasmid, the mRNA and protein level of DNMTs were detected by QRT-PCR (**D**, **E**) and Western blotting (**F**). * *P* ˂ 0.05, ** *P* ˂ 0.01. (**G**) Expression level of DNMT3A in tumor is higher than in normal tissue, shown by GEPIA database. (**H**) The correlation between SYNCRIP and DNMT3A in colorectal cancer.

We then sought to investigate the mechanism of how SYNCRIP regulated the CRC cell proliferation and motility. DNMT was highly expressed in CRC and the expression level of SYNCRIP significantly correlated with the reduced survival probability^30^. In addition, SYNCRIP was reported to partner with Lin28a and regulate the expression of DNMT3A^31^. Thus, we wonder whether SYNCRIP could regulate DNMT expression in CRC cells. SYNCRIP depletion significantly decreased the mRNA and protein level of DNMT3A, but not DNMT1 or DNMT3B in both SW480 and HCT116 cells (Figs. 5A–C, S4A). On the contrary, overexpression of SYNCRIP significantly increased the expression of DNMT3A, but not DNMT1 or DNMT3B (Figs. 5D–F, S4B). More importantly, we found that the expression level of DNMT3A in colorectal tumor (n = 275) is higher than in normal tissue (n = 41) by analyzing TCGA database (Fig. 5G). The CRC patients with higher DNMT3A expression showed lower overall survival rate (Fig. S4C). Furthermore, the correlation between the expression of SYNCRIP and DNMT3A was studied by linear regression analysis of data from the TCGA database. A significant positive association between the expression of SYNCRIP and DNMT3A in CRC was identified (P = 2.6e−10), and the R^2^ coefficient was 0.37 (Fig. 5H), which suggested a moderate correlation between SYNCRIP and DNMT3A.

**Figure 6:**
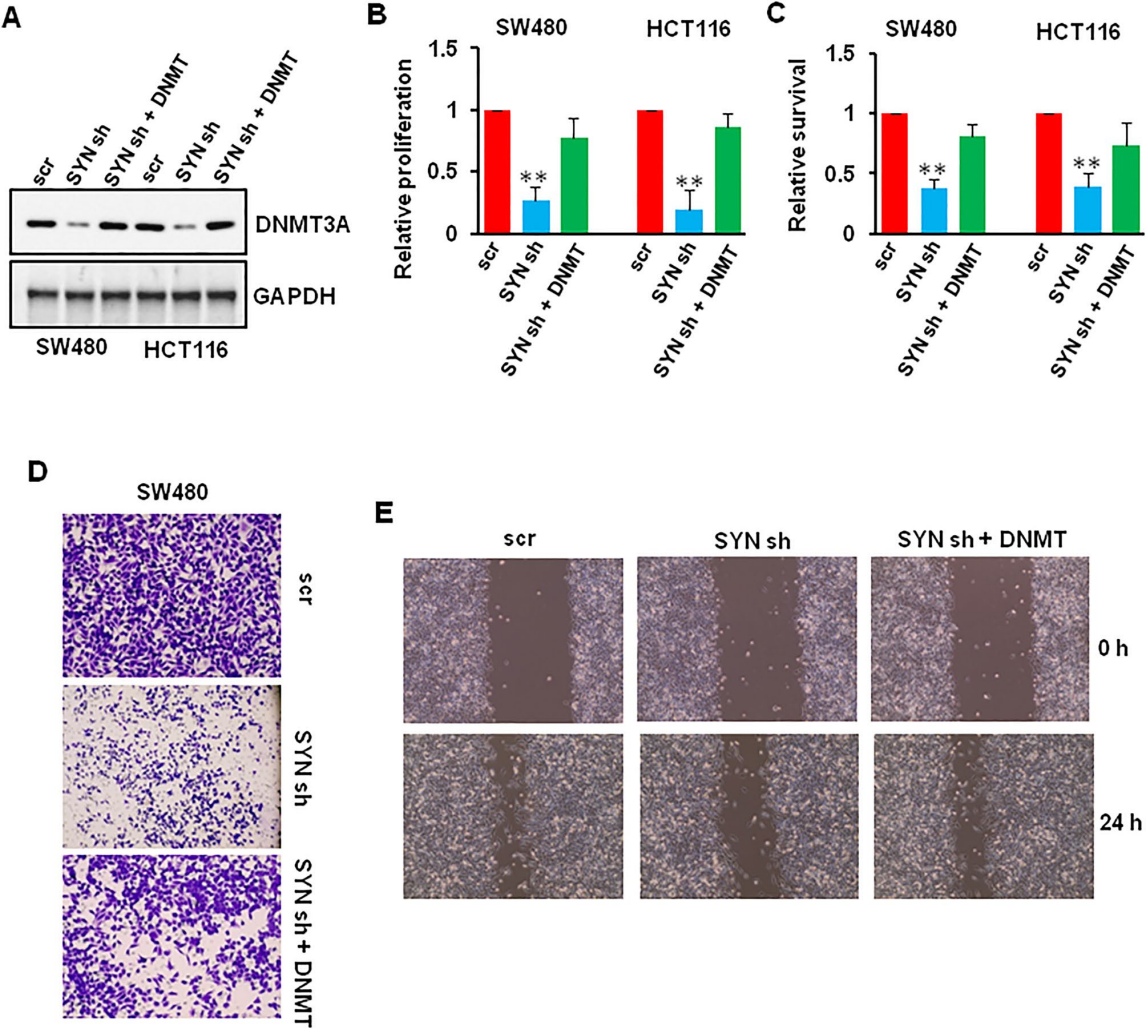
SYNCRIP regulated colorectal cancer cell growth and migration via DNMT3A. (**A**) SW480 and HCT116 cells were infected with scr or SYNCRIP shRNA followed with or without DNMT3A plasmid, the protein level of DNMT3A was detected by Western blotting. (**B**, **C**) SW480 and HCT116 cells were infected with scr or SYNCRIP shRNA followed with or without DNMT3A plasmid, the proliferation and survival of SW480 and HCT116 cells were measured using CCK-8 assay (**B**) and trypan blue stain (**C**). ** *P* ˂ 0.01. (**D**, **E**) SW480 and HCT116 cells were infected with scr or SYNCRIP shRNA followed with or without DNMT3A plasmid, the motility of SW480 and HCT116 cells were analyzed using migration assay (**D**) and wound healing assay (**E**).

Since we have found that SYNCRIP regulated the expression of DNMT3A, we wonder whether SYNCRIP regulated CRC cell proliferation and motility via DNMT3A. We knockdown SYNCRIP using shRNA followed with or without DNMT3A overexpression in SW480 and HCT116 cells (Figs. 6A, S5A). Consistent with our previous finding (Fig. 3), SYNCRIP depletion significantly inhibited CRC cell proliferation and motility. However, DNMT3A overexpression blocked the inhibitory effect of SYNCRIP depletion (Figs. 6B–E, S5B,C), which suggested that SYNCRIP regulated CRC cell proliferation and motility via DNMT3A.

### SYNCRIP regulated the expression of p16 via regulating DNMT3A expression

**Figure 7:**
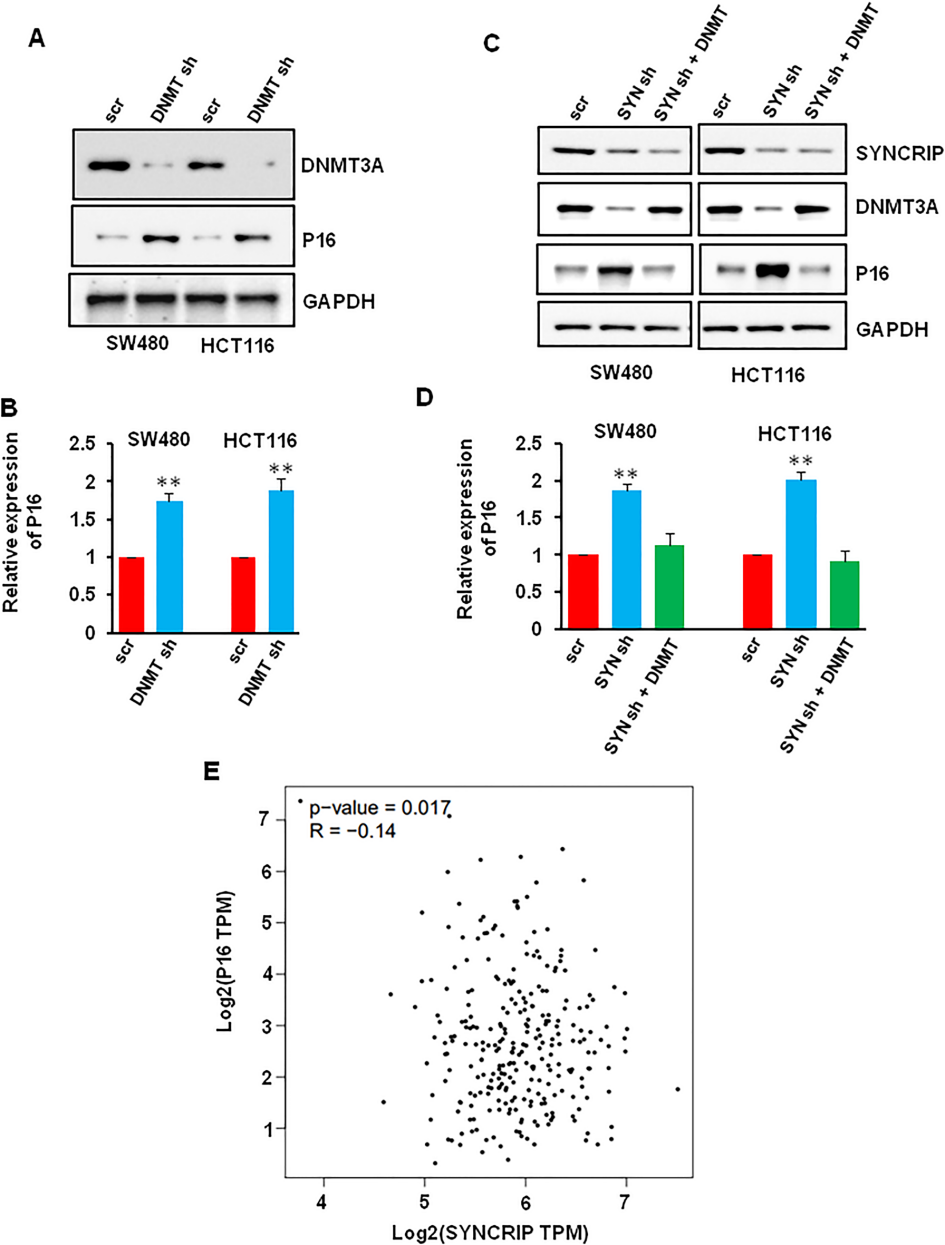
SYNCRIP regulated the expression of p16 via DNMT3A. (**A**, **B**) SW480 and HCT116 cells were infected with scr or DNMT3A shRNA for 72 h, the protein and mRNA level of p16 were detected by Western blotting (**A**) and QRT-PCR (**B**). **P ˂ 0.01. (**C**, **D**) SW480 and HCT116 cells were infected with scr or SYNCRIP (shRNA) shRNA followed with or without DNMT3A plasmid, the protein and mRNA level of p16 were detected by Western blotting (C) and QRT-PCR (**D**). **P ˂ 0.01. (**E**) The correlation between SYNCRIP and p16 in colorectal cancer.

DNMTs could suppress the expression of tumor suppressor genes in human cancer cells^32^. P16 is one of the most common studied tumor suppressor genes, and is regulated by DNMT3s in cervical cancer^33^. To confirm that DNMT3A could regulate the expression of p16 in colorectal cancer, we knockdown the DNMT3A in SW480 and HCT116 cells. The protein and mRNA level increased after DNMT3A depletion (Figs. 7A,B, S6A). Not surprisingly, the expression of p16 increased after SYNCRIP depletion. However, DNMT3A overexpression blocked the effect of SYNCRIP depletion on p16 expression, which indicated that SYNCRIP regulated the expression of p16 via DNMT3A (Figs. 7C,D, S6B). Furthermore, the linear regression analysis of data from the TCGA database indicated negative correlation between SYNCRIP and p16 (Fig. 7E).

### SYNCRIP regulated colorectal cancer cell growth in vivo

**Figure 8:**
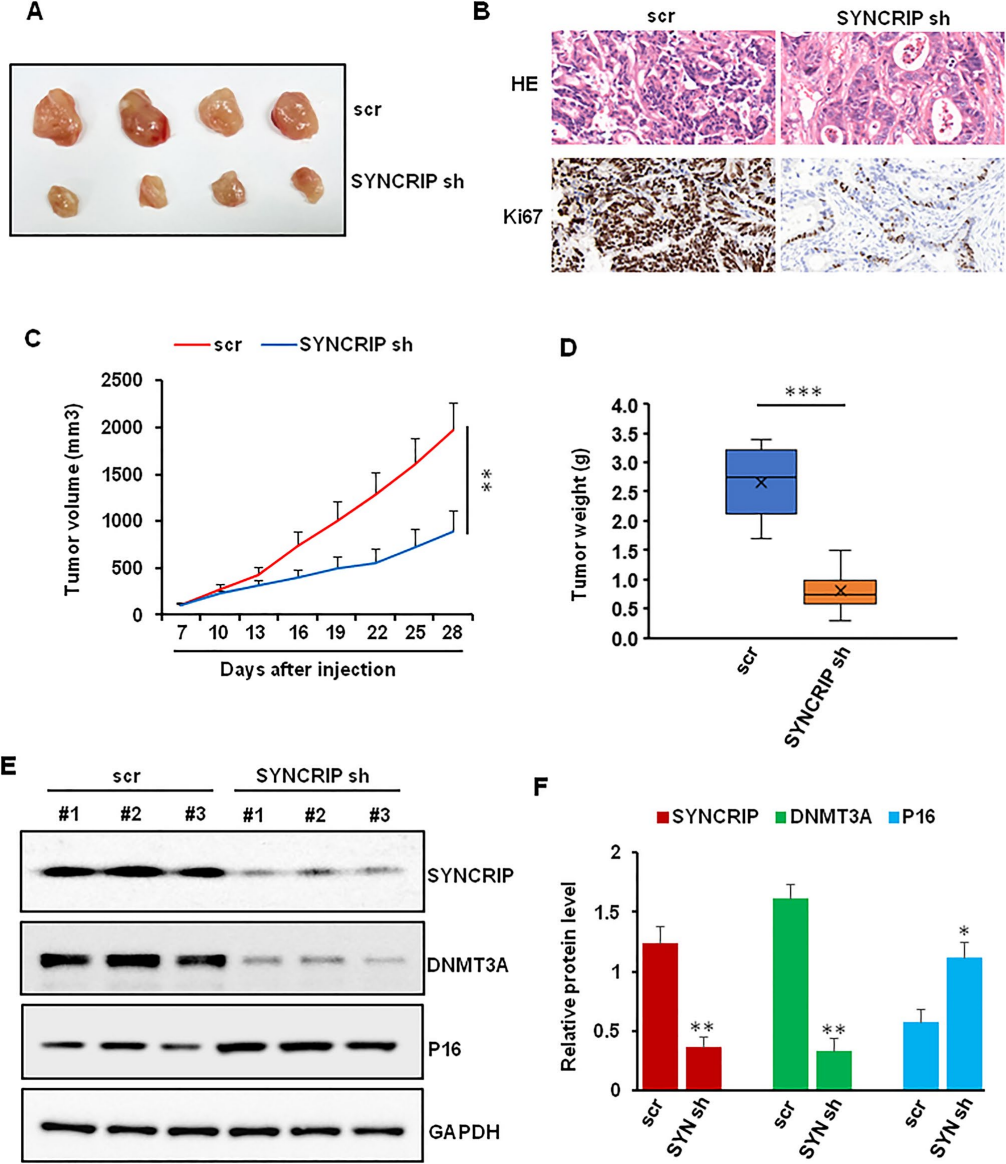
Effects of SYNCRIP depletion on tumor growth in vivo. (**A**) Typical photos of tumors on day 28 from scr and on shRNA groups. (**B**) HE and Ki67 staining of sections from xenograft tumors. (**C**, **D**) SYNCRIP depletion significantly decreased the tumor size (**C**) and weight (**D**). **P ˂ 0.01, ***P ˂ 0.001. (**E**, **F**) Protein level of SYNCRIP and p16 in 3 representative tumors from each group were analyzed by western blotting (**E**) and quantified by densitometry analysis (**F**). *P ˂ 0.05, **P ˂ 0.01.

To further confirm SYNCRIP as a therapeutic target, we tested the function of SYNCRIP on colorectal tumor cell growth in a mouse xenograft model. SW480 cells were infected with scramble or SYNCRIP shRNA before injected into NOD/SCID mice frank. At the end of the experiments (4 weeks), the mice were euthanized, and the weight of tumor was analyzed. As shown in Fig. 8A–D, the tumor size and weight were significantly decreased in SYNCRIP depletion group compared with control group. Moreover, immunohistochemical staining showed that the cell proliferation indicator Ki67 of tumors was decreased in the SYNCRIP depletion group. The effect of SYNCRIP depletion on DNMT3A, DNMT3B, and p16 expression were evaluated. As shown in Fig. 8E and F, the protein level of DNMT3A and p16 were lower, while p16 level is higher in SYNCRIP depletion group compared with scramble control group.

## Discussion

As a group of RNA-binding proteins (RBPs), hnRNPs are important for gene expression regulation and many of the hnRNPs are keystones in tumor development. The functions of hnRNPs rely on their cellular localization. Most of the hnRNPs are predominantly present in the nucleus and possess a conventional nuclear localization signal^6^. hnRNP proteins undergo several post-translational modifications, and such changes regulate their subcellular localization and biological activity. Reported post-translational modifications on hnRNPs include phosphorylation, simulation, ubiquitination, and methylation^34^. For example, MAPK/ERK phosphorylated hnRNP K, which in turn change its cellular localization and ability to silence mRNA translation^35^. Except post-translational modification, the expression of SYNCRIP could be regulated by oncogenic transcription factor, such as c-Myc. C-Myc upregulates the expression of hnRNPs, which ensuring a high PKM2/PKM1 ratio in cancer cells^36^. Another oncoprotein, KITENIN promotes aerobic glycolysis by upregulating c-myc/hnRNP axis in colorectal cancer^37^. In addition, lncNT5E enhanced the activity of SYNCRIP promoter to promote its transcription in pancreatic cancer^38^. The downstream targets of hnRNPs include multiple oncogenes. The hnRNP A1 has been associated with tumor cell growth and metastasis in several different cancer types. Several oncogenes, including *Kirsten rat sarcoma viral oncogene homolog* (*KRAS*), *Harvey rat sarcoma viral oncogene homolog* (*HRAS*) and a splice variant of *Recepteur d’Origine Nantais* (Δ*RON*), have been identified as direct targets of hnRNP A1^39^. hnRNP C was also reported to regulate another oncogene named *breast cancer* (*BRCA*)^40^. Its role in gene expression regulation, either on transcriptional or translational level, makes hnRNP E one of the best-studied hnRNP in cancer research. hnRNP E1 together with hnRNP E2 regulated the half-life of *p21*^*WAF*^ and therefore regulate the cell cycle in a p53-independent manner^41^. hnRNP E1 could also stabilized the mRNA of p63, another transcription factor and a p53 family protein^42^. Unlike other hnRNPs, hnRNP Q (also known as SYNCRIP) was less associated with cancer. However, two studies showed that one of the hnRNP Q isoform, hnRNP Q1, is involved in tumorigenesis via regulating cell cycle-related genes, such as Aurora-A in colorectal cancer^18,19^, which suggested SYNCRIP as a key player in colorectal cancer development and pathology. We first analyzed the expression of SYNCRIP in TCGA and GEPIA database and found that the expression of SYNCRIP is significantly higher in CRC tumor compared with normal tissue. These finding was further confirmed by comparing the expression of SYNCRIP from tumor tissue and adjacent normal tissues, normal human colon mucosal epithelial cell line and CRC cell lines. More importantly, SYNCRIP depletion inhibited CRC cell growth and migration, whereas SYNCRIP overexpression promoted CRC cell growth and migration. These results suggested SYNCRIP as an oncogene in colorectal cancer.

Accumulating evidence suggests that aberrant DNA methylation was a driver of many human malignant tumor types^43,44^. DNA methylation is an epigenetic modification typically occurred in the CpG islands of the gene promoter region and is catalyzed by a family of DNA methyltransferases (DNMTs). This modification will generate highly stable and transmittable gene silencing^45^. There are three subtypes of DNMTs in mammals, DNMT1, DNMT3A, and DNMT3B. Among the three DNMTs, DNMT1 prefers to methylate hemi-methylated DNA to maintain the existing DNA methylation, while DNMT3A and DNMT3B are de novo methyltransferases that apply to unmethylated DNA, and are critical enzymes during embryogenesis and pathogenesis^46^. Many studies have shown that dysregulation of DNMT3A leads to oncogenesis in several cancer types, including gastric cancer, hepatocellular cancer, and colorectal cancer^26,27,47^. To investigate the mechanism of how SYNCRIP regulated the CRC cell proliferation and motility, we investigated the effect of SYNCRIP on DNMTs expression. Surprisingly, SYNCRIP only regulated the expression of DNMT3A, but not DNMT1 and DNMT3B. Also, a significant positive correlation between the expression of SYNCRIP and DNMT3A in CRC was identified. More importantly, DNMT3A overexpression blocked the inhibitory effect of SYNCRIP depletion on CRC cell growth and migration, which suggested that SYNCRIP regulated CRC cell tumorigenesis via DNMT3A. However, the mechanism of how SYNCRIP regulates the expression of DNMT3A remains unclear. Indeed, an important binding partner of SYNCRIP, Lin28a, was proved to bind *Dnmt3a* mRNA and regulate the expression of DNMT3A protein^31^. More importantly, the activity of Lin28a is dependent on its binding partner, such as SYNCRIP^31^, which suggested that SYNCRIP could regulate the expression of DNMT3A through Lin28a.

The incidence of colorectal cancer (CRC) is increasing globally and has an extremely high mortality rate. Activation of oncogenes and inactivation of tumor suppressor genes contribute to the manipulation of CRC malignant phenotypes. P16 is one of the most common studied tumor suppressor genes. The promoter regions of p16 are often methylated, which decreases the levels of p16. Previous studies have demonstrated that *p16* promoter hypermethylation was found in 26% of CRC cases^48^. In cervical cancer, DNMT3A and DNMT3B regulated the methylation of p16 promoter, and affected its expression^33^. Thus, we wonder whether DNMT3A could regulate p16 expression in CRC cells. We found that DNMT3A depletion increased the expression of p16, which showed same results as SYNCRIP depletion. More importantly, DNMT3A overexpression blocked the effect of SYNCRIP depletion on p16 expression, which indicated that SYNCRIP regulated p16 expression via DNMT3A.

Taken all together, we investigated the important role of SYNCRIP in regulating colorectal cancer growth and motility in the present study. We found that SYNCRIP regulated the expression of DNMT3A, which then methylated the promoter of tumor suppressor-p16 and inhibited its expression. Our results suggested SYNCRIP as a potential prognostic biomarker and therapeutic target in colorectal cancer.

## Supplementary Information

Supplementary Figures.

Supplementary Information 1.

## Data Availability

All data generated or analyzed during this study are included in this published article and its supplementary information files.
