# Supplementary material for: The up-regulation of SYNCRIP promotes the proliferation and tumorigenesis via DNMT3A/p16 in colorectal cancer
Source: Sci Rep. 2024 Sep 16;14:21570. doi: 10.1038/s41598-024-59575-6 (PMC11405714; doi:10.1038/s41598-024-59575-6)
Supplement: Supplementary file 1 — Supplementary Figures. [file 41598_2024_59575_MOESM1_ESM.pdf]

Supplementary Fig. 1

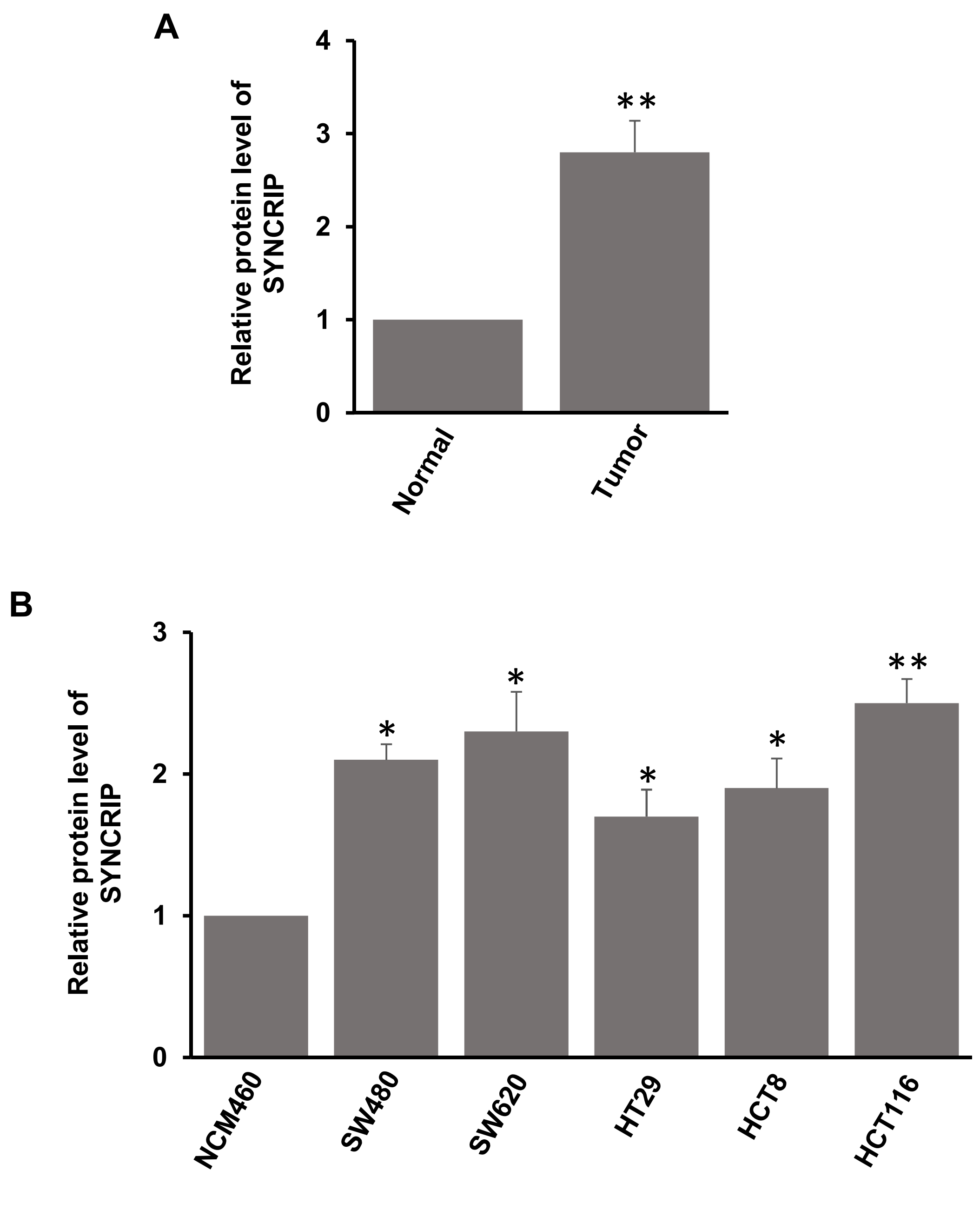

Figure S1. The expression of SYNCRIP in colorectal cancer cells.

(A) Relative protein level of SYNCRIP in CRC tissues and normal tissues. (B) The relative protein level of SYNCRIP in human normal colon mucosal epithelial cell and colorectal cancer cell lines. * $P < 0.05$, ** $P < 0.01$.

Supplementary Fig. 2

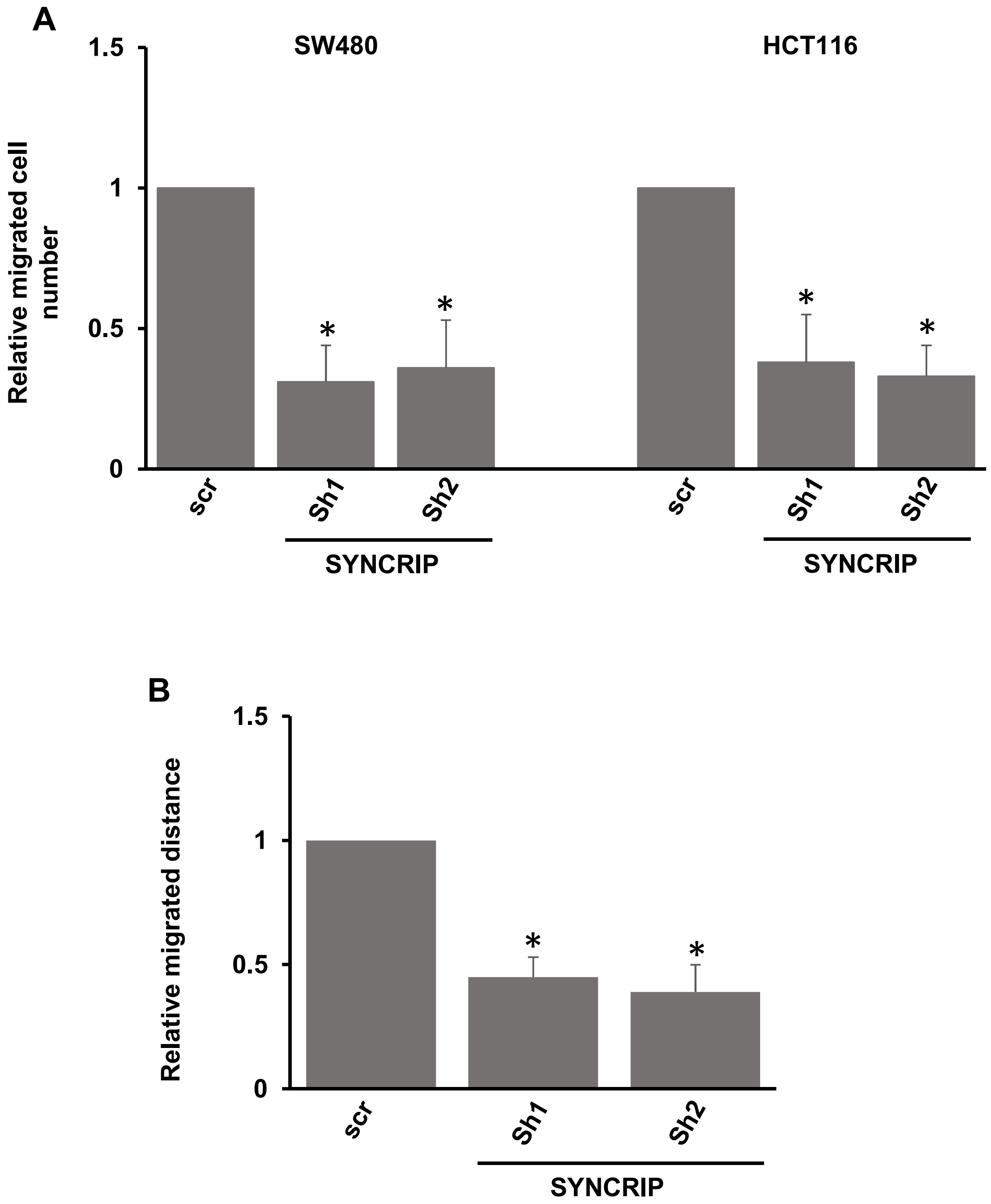

Figure S2. The effect of SYNCRIP depletion on cell motility.

(A) Relative migrated cells after SYNCRIP knockdown. (B) Relative migrated distance after SYNCRIP knockdown. * $P < 0.05$.

Supplementary Fig. 3

A

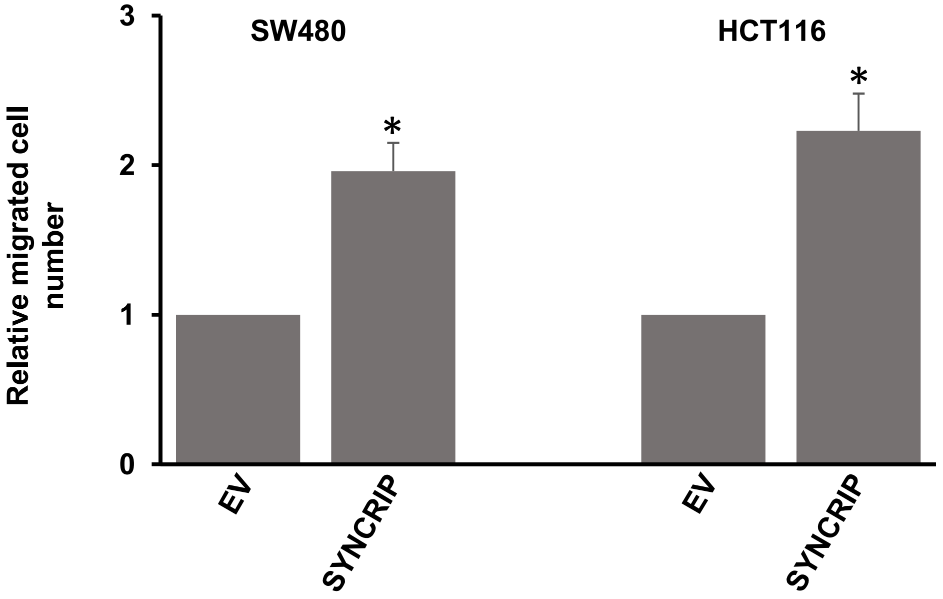

B

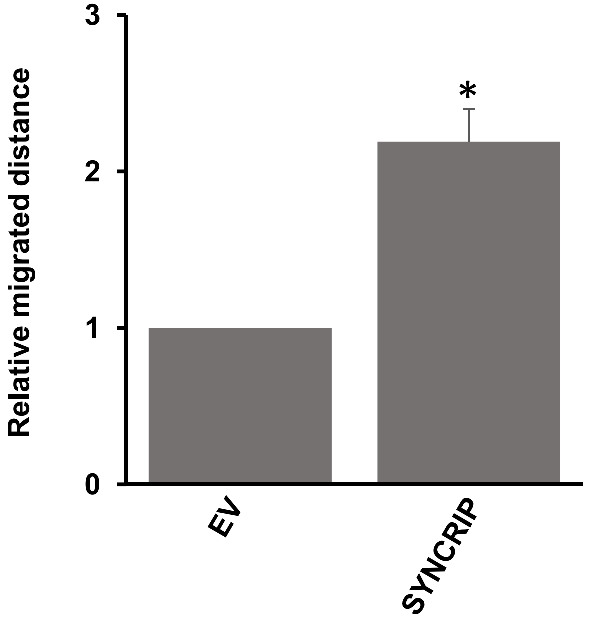

Figure S3. The effect of SYNCRIP overexpression on cell motility.

(A) Relative migrated cells after SYNCRIP overexpression. (B) Relative migrated distance after SYNCRIP overexpression. * $P < 0.05$.

Supplementary Fig. 4

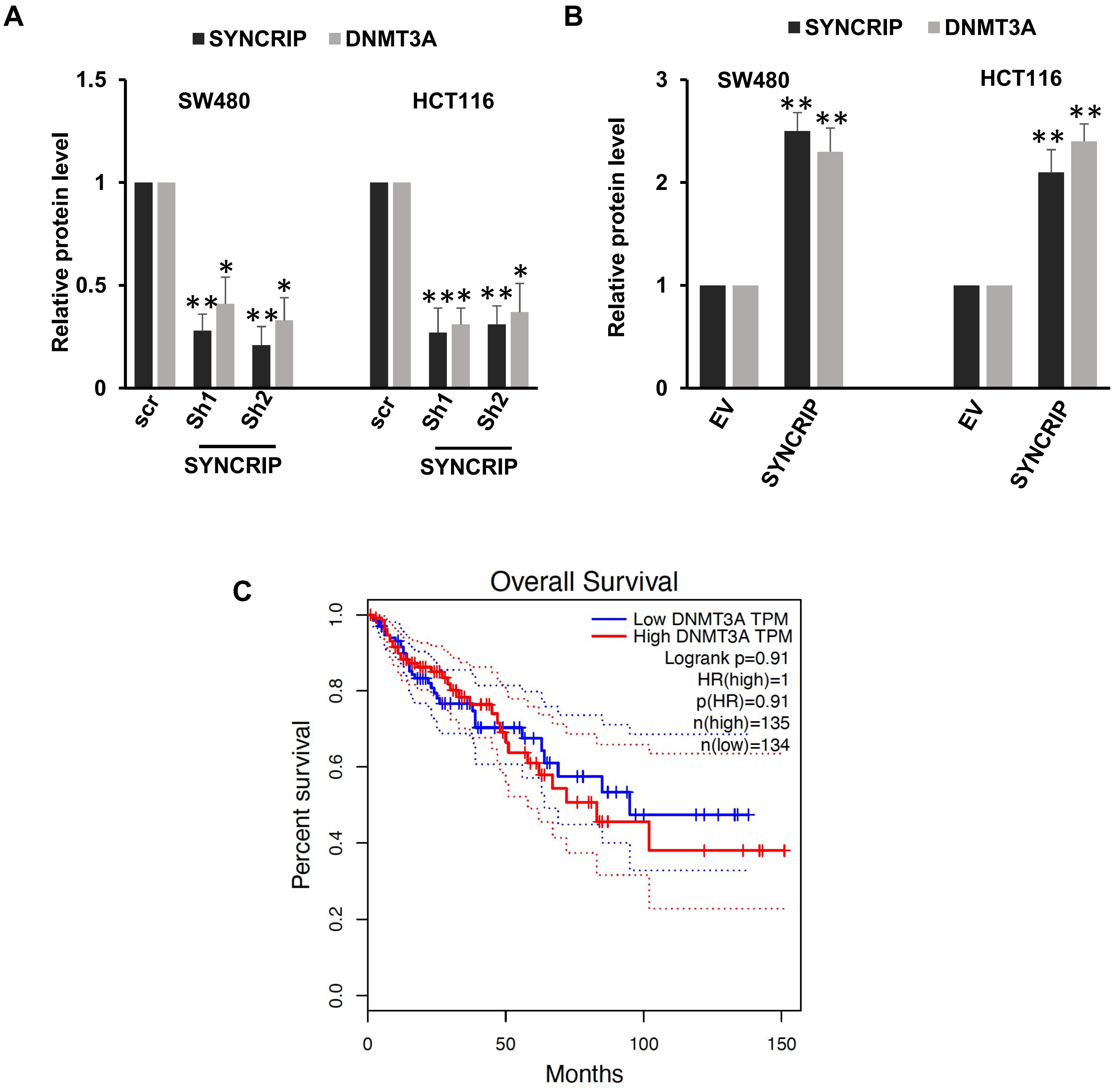

Figure S4. The effect of SYNCRIP on regulating DNMT3A expression.

(A) Relative protein level of SYNCRIP and DNMT3A after SYNCRIP depletion. (B) Relative protein level of SYNCRIP and DNMT3A after SYNCRIP overexpression. (C) The overall survival rate of patients with high or low level of DNMT3A. * $P < 0.05$, ** $P < 0.01$.

Supplementary Fig. 5

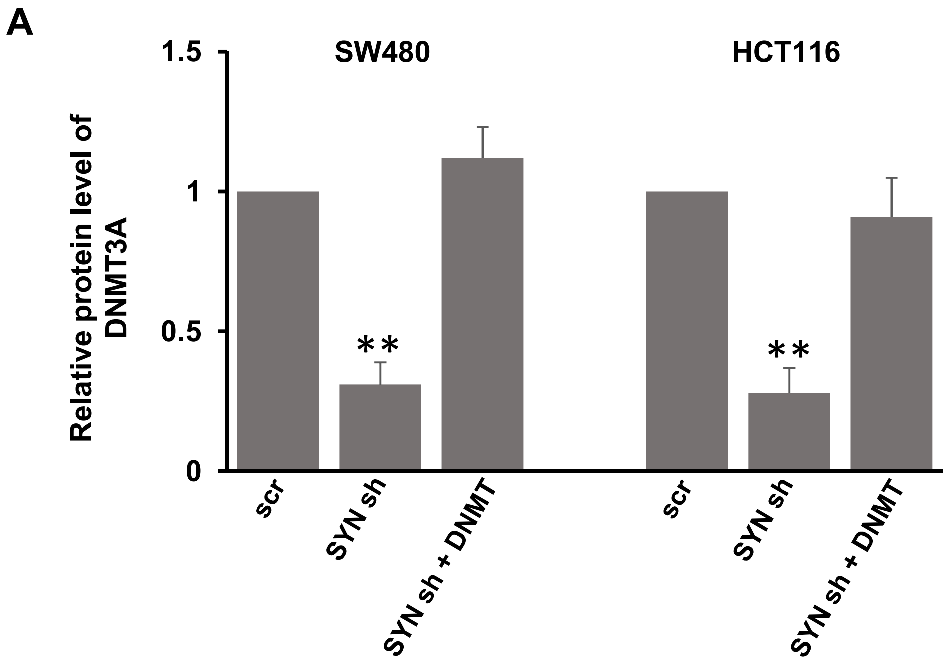

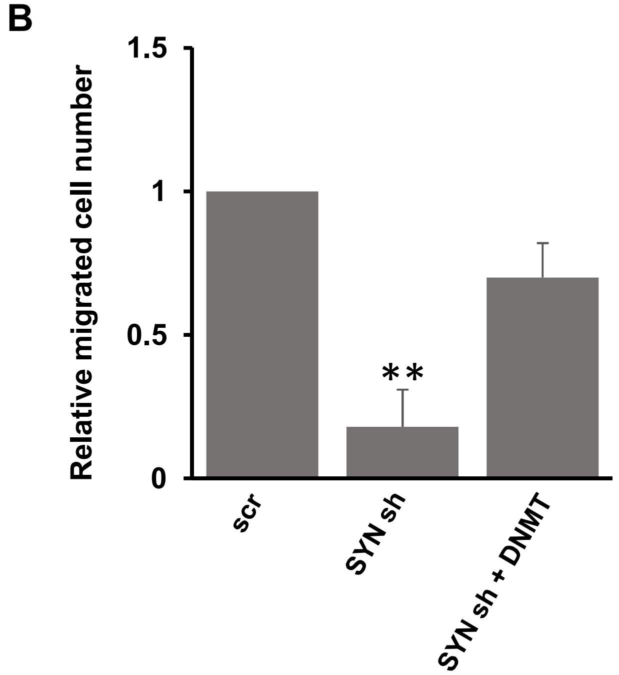

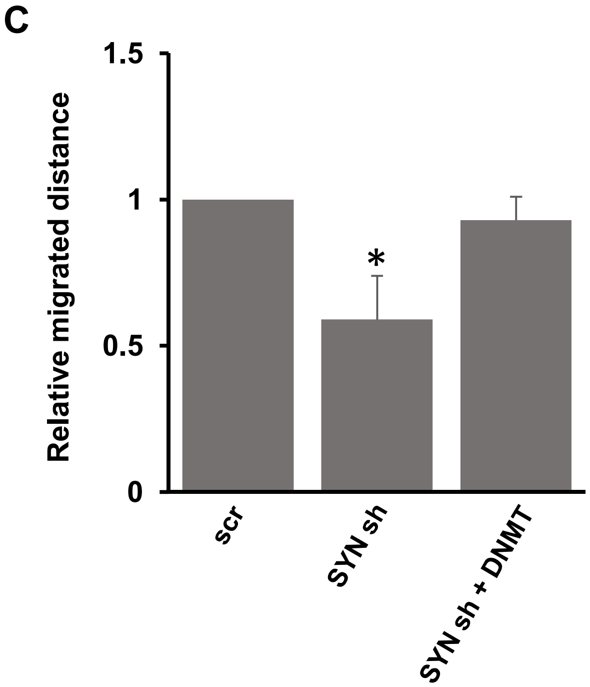

Figure S5. SYNCRIP regulated colorectal cancer cell migration via DNMT3A.

(A) Relative protein level of DNMT3A after SYNCRIP depletion followed with or without DNMT3A overexpression. (B, C) Relative migrated cells and distance after SYNCRIP depletion followed with or without DNMT3A overexpression. * $P < 0.05$, ** $P < 0.01$.

Supplementary Fig. 6

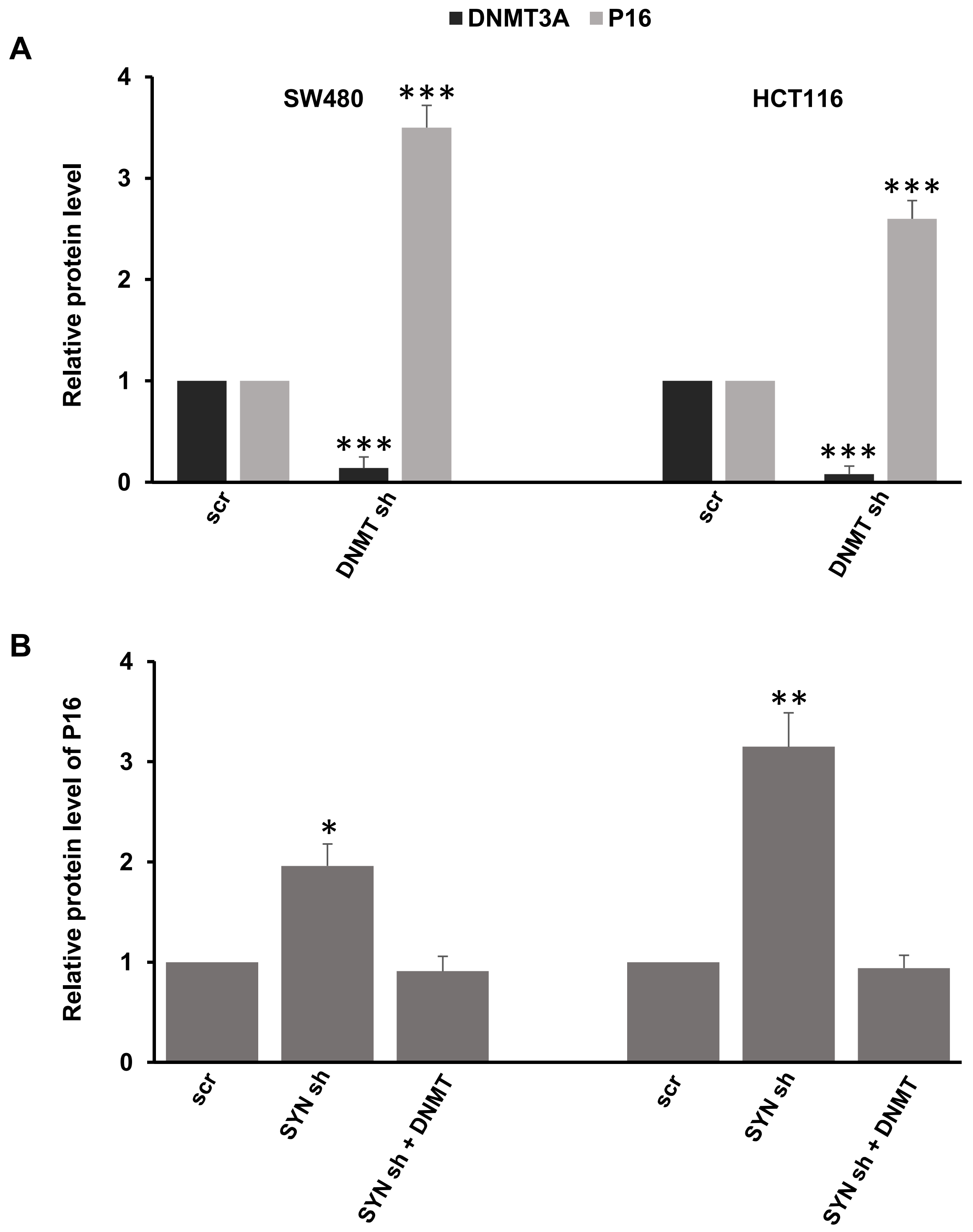

Figure S6. SYNCRIP regulated the expression of p16 via DNMT3A.

(A) Relative protein level of DNMT3A and P16 after DNMT3A depletion . (B) Relative protein level of P16 after SYNCRIP depletion followed with or without DNMT3A overexpression. * *P* < 0.05, ** *P* < 0.01 , *** *P* < 0.001.
