# Supplementary material for: The up-regulation of SYNCRIP promotes the proliferation and tumorigenesis via DNMT3A/p16 in colorectal cancer
Source: Sci Rep. 2024 Sep 16;14:21570. doi: 10.1038/s41598-024-59575-6 (PMC11405714; doi:10.1038/s41598-024-59575-6)

Fig. 2B

Fig. 2B, SYNCRIP

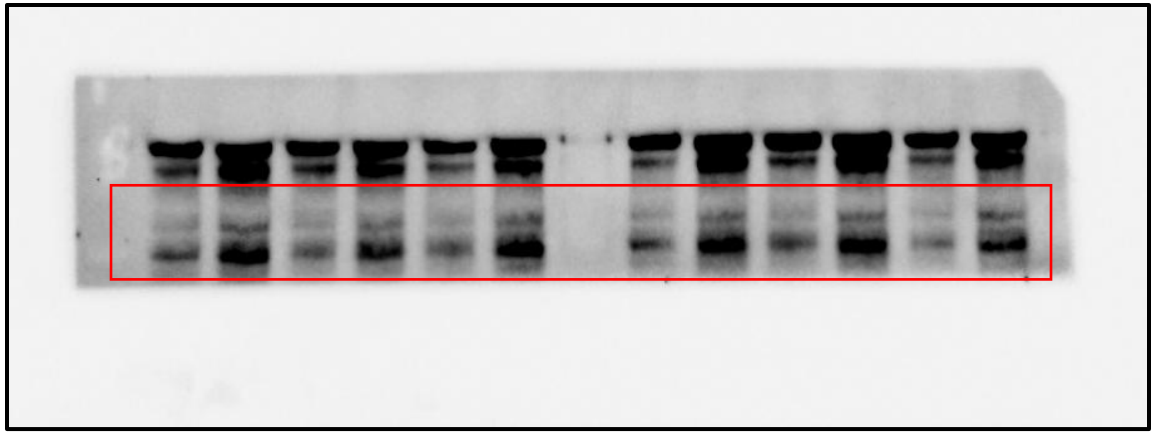

Fig. 2B, GAPDH

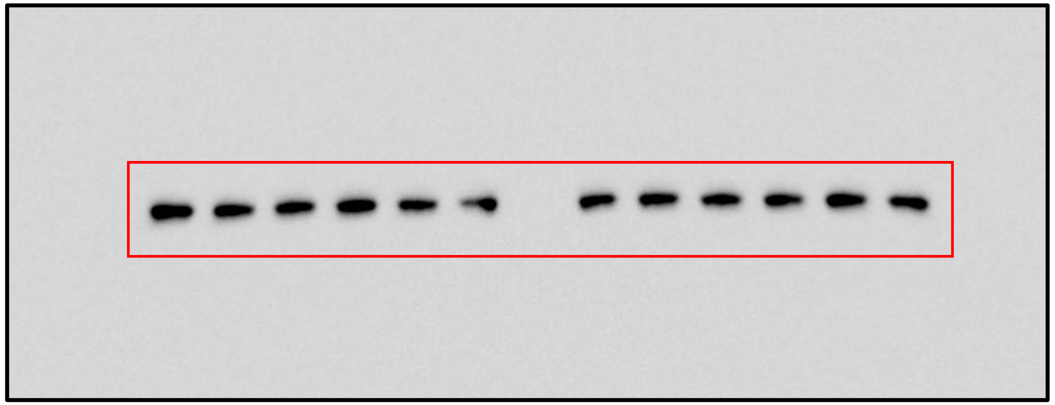

Fig. 2D

Fig. 2D, SYNCRIP

Fig. 2D, GAPDH

n=1

n=2

n=3

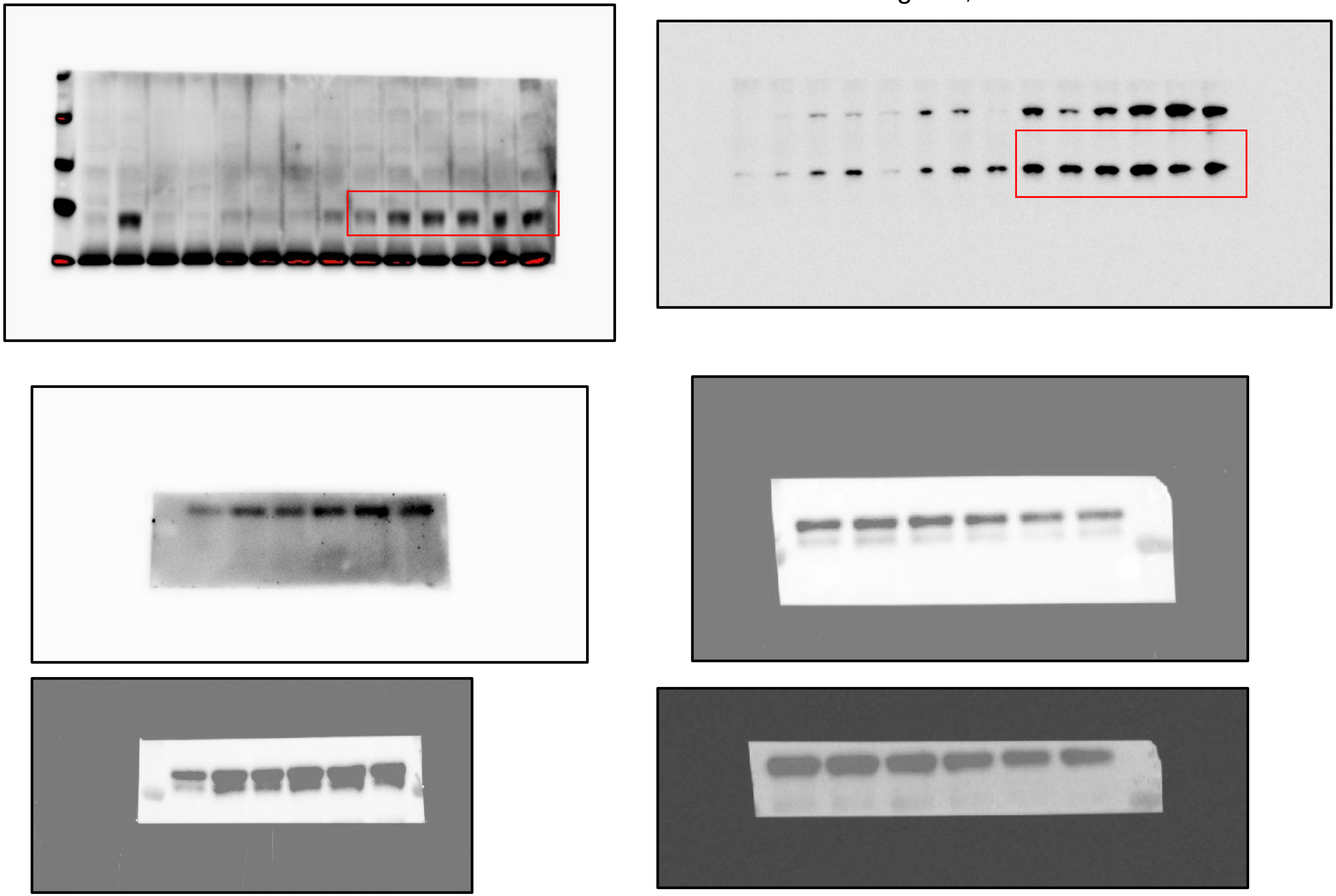

Fig. 5C **n=1**

Fig. 5C, SYNCRIP

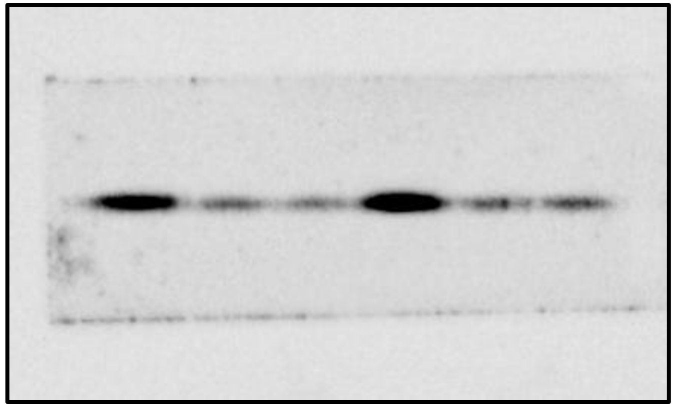

Fig. 5C, DNMT1

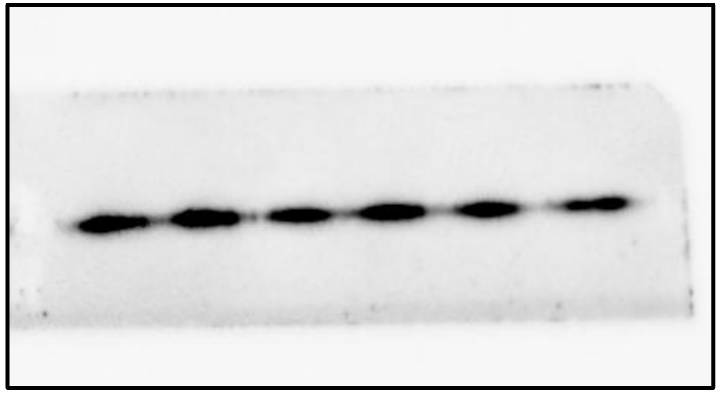

Fig. 5C, DNMT3A

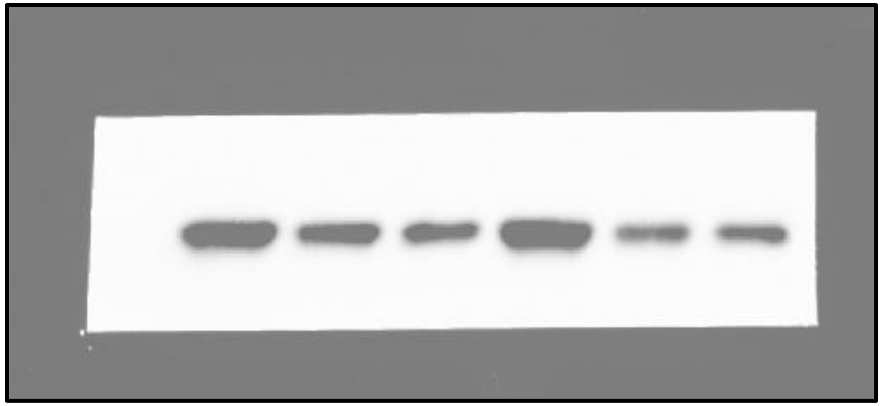

Fig. 5C, DNMT3B

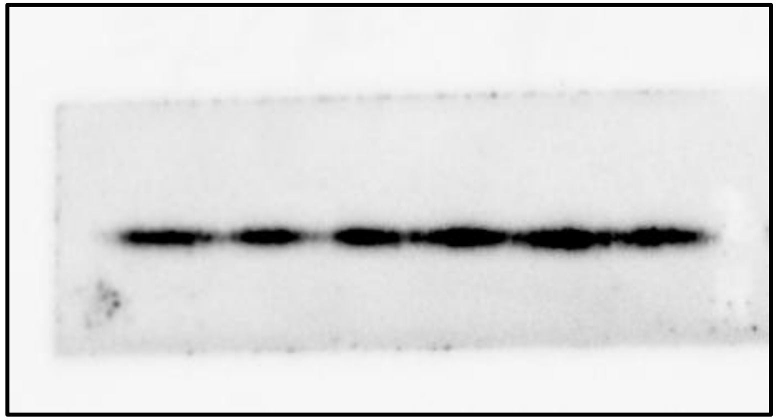

Fig. 5C, GAPDH

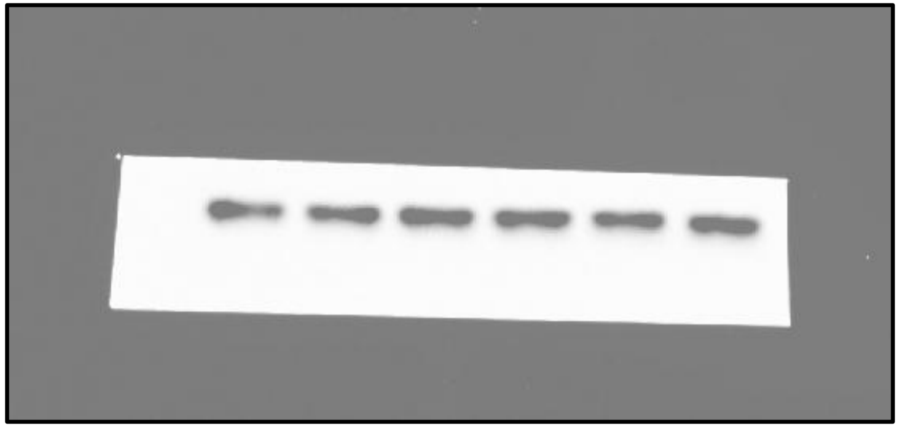

Fig. 5C n=2

Fig. 5C, SYNCRIP

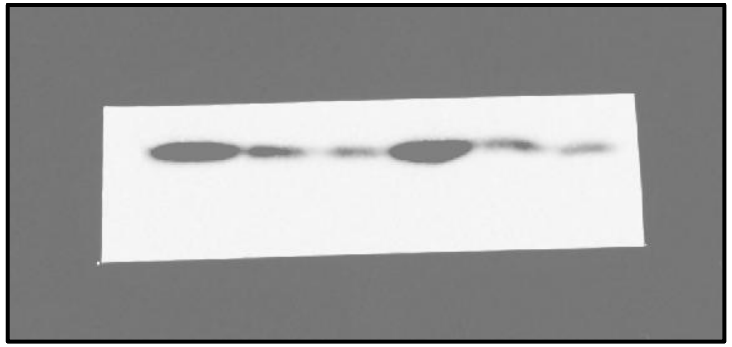

Fig. 5C, DNMT1

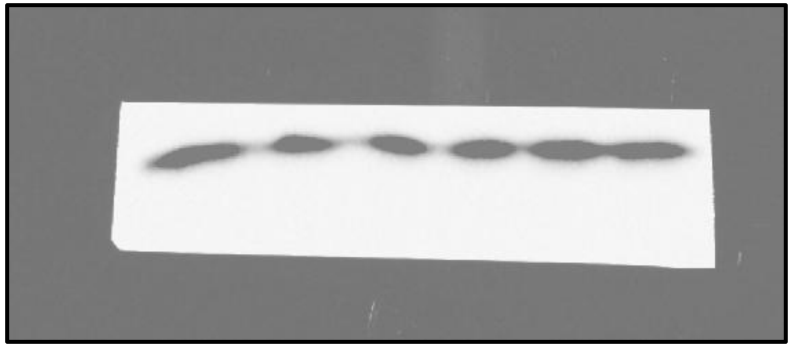

Fig. 5C, DNMT3A

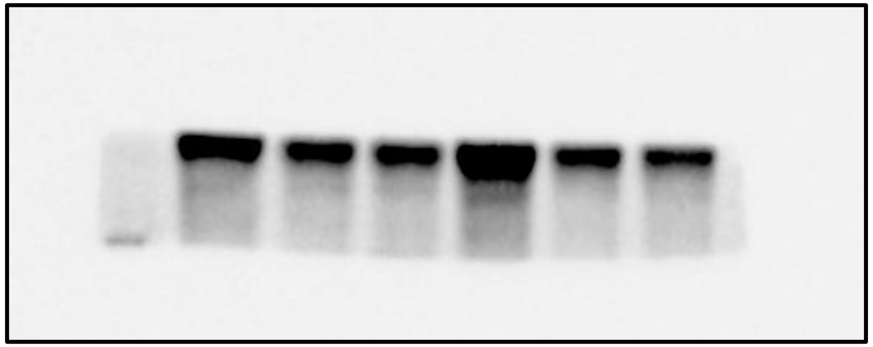

Fig. 5C, DNMT3B

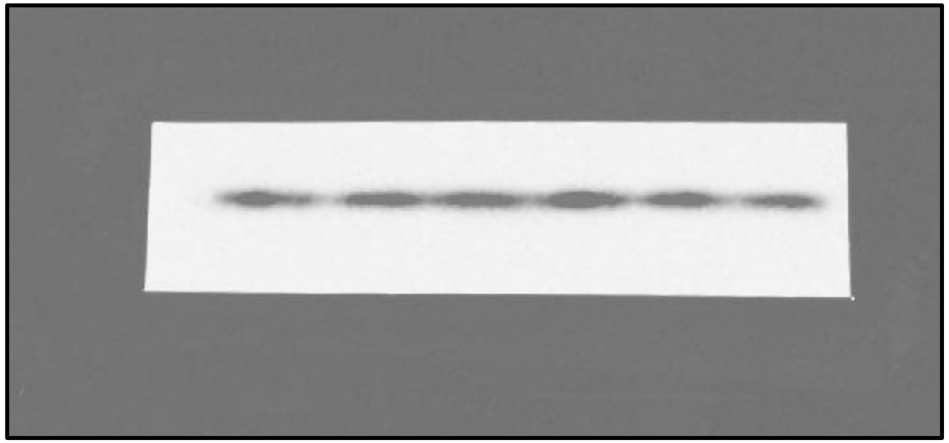

Fig. 5C, GAPDH

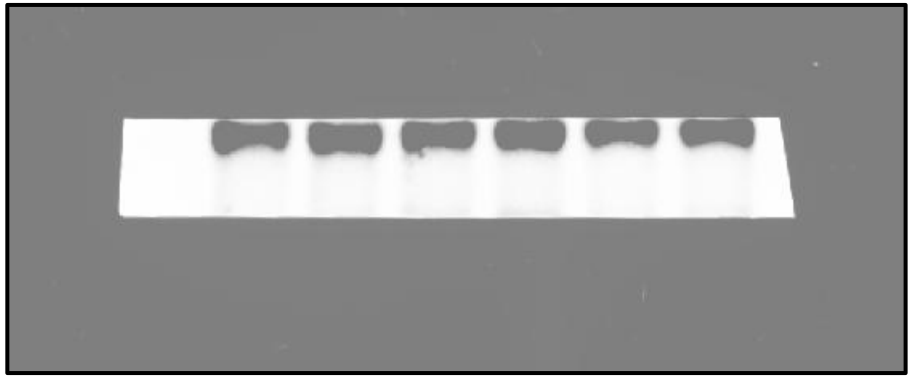

Fig. 5C n=3

Fig. 5C, SYNCRIP

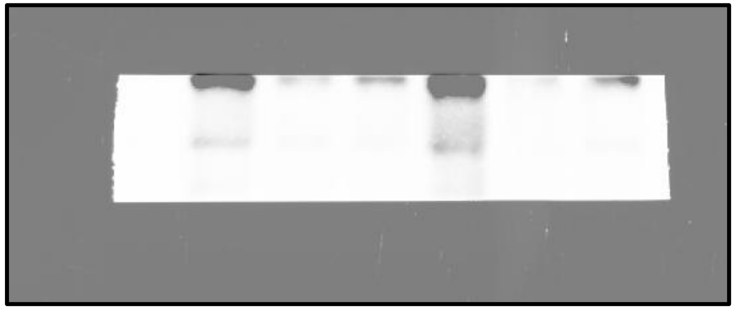

Fig. 5C, DNMT1

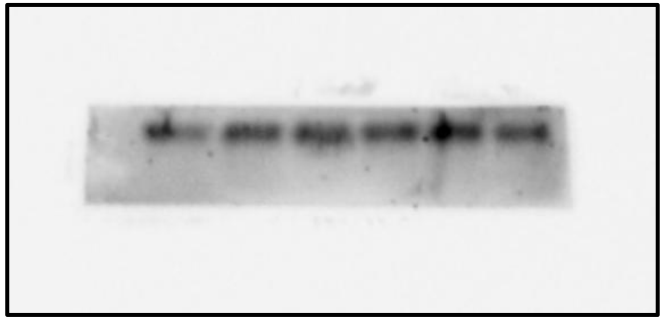

Fig. 5C, DNMT3A

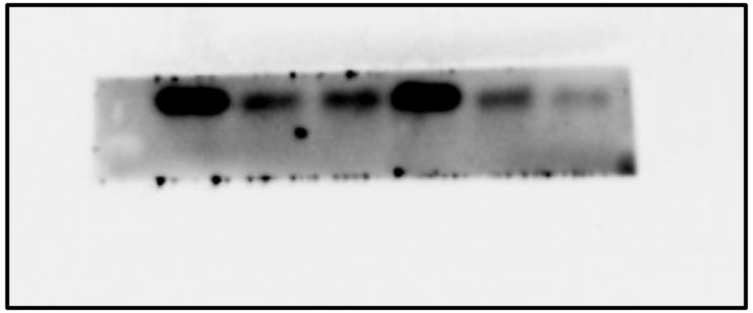

Fig. 5C, DNMT3B

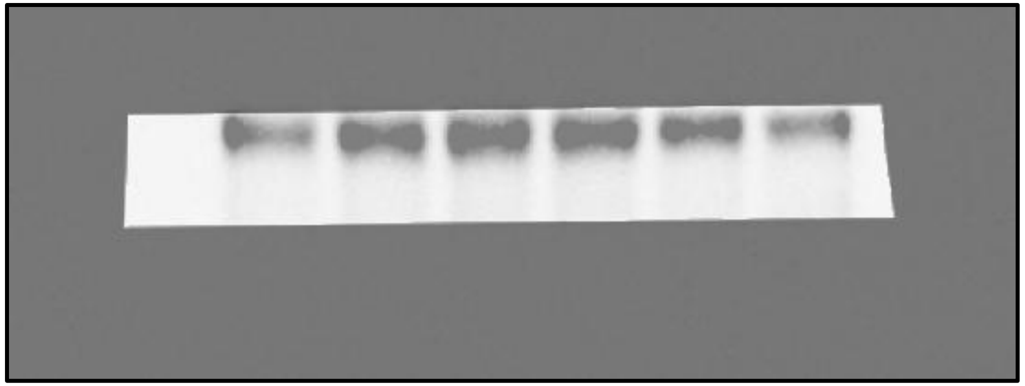

Fig. 5C, GAPDH

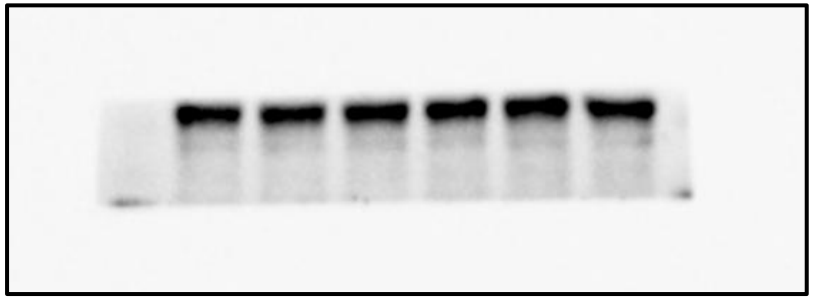

Fig. 5F **n=1**

Fig. 5F, SYNCRIP

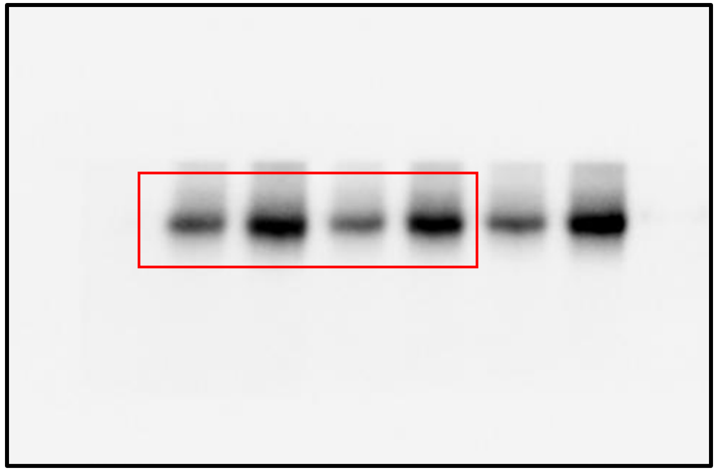

Fig. 5F, DNMT1

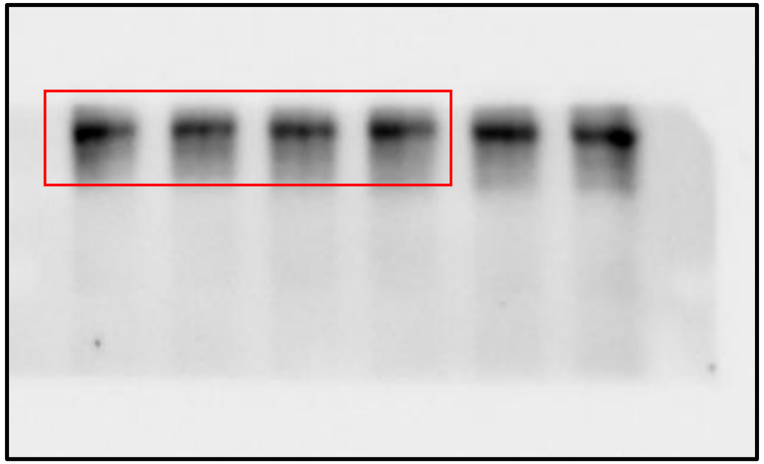

Fig. 5F, DNMT3A

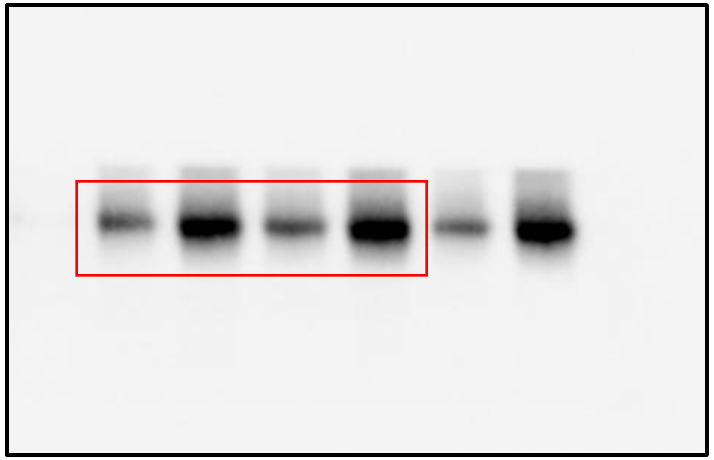

Fig. 5F, DNMT3B

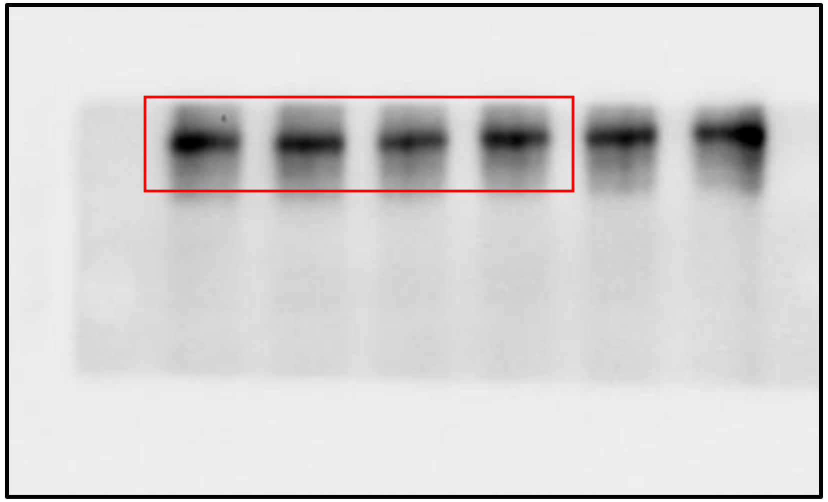

Fig. 5F, GAPDH

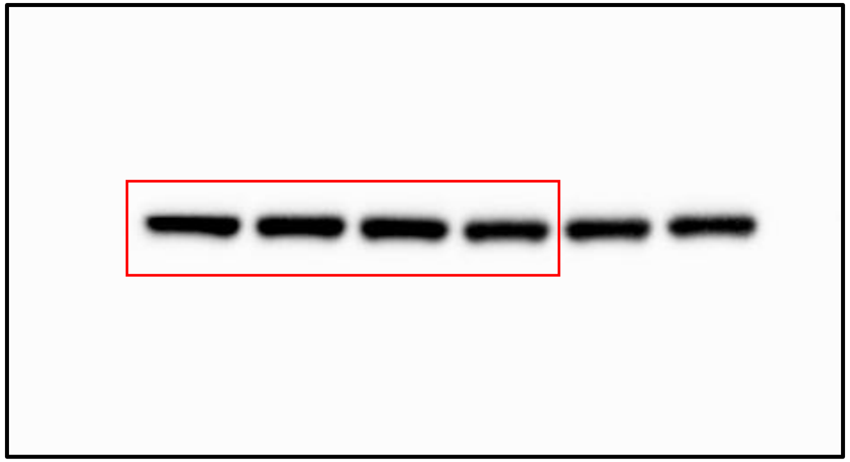

Fig. 5F n=2

Fig. 5F, SYNCRIP

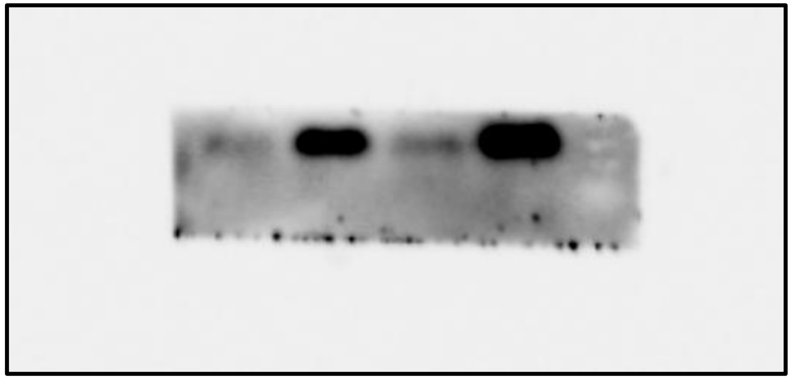

Fig. 5F, DNMT1

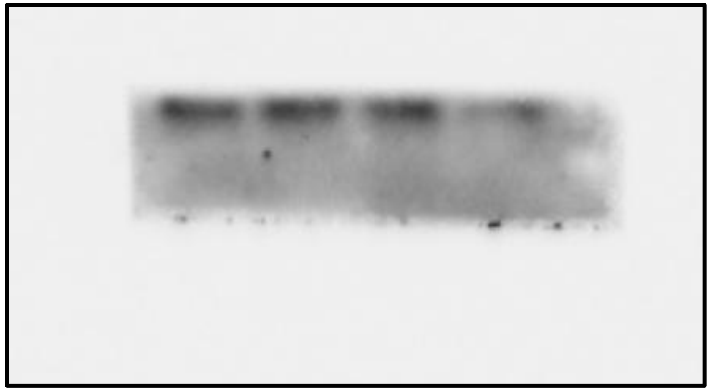

Fig. 5F, DNMT3A

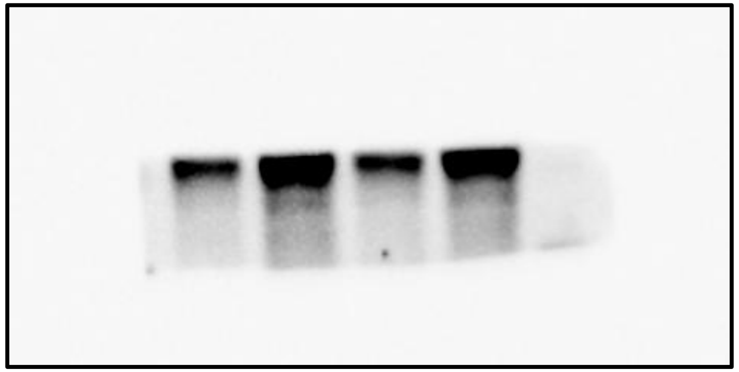

Fig. 5F, DNMT3B

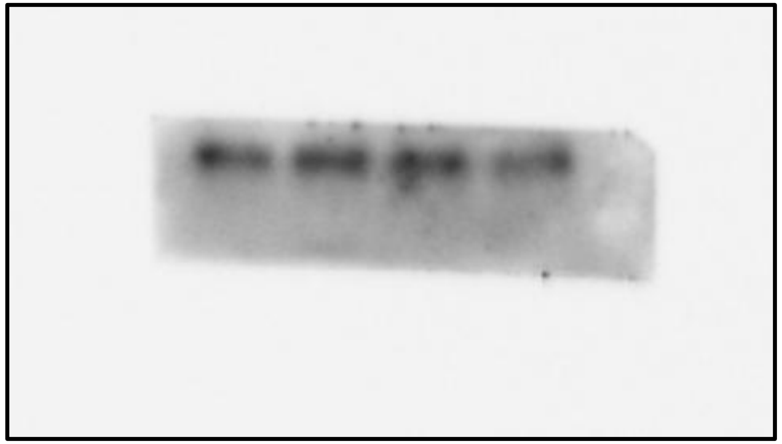

Fig. 5F, GAPDH

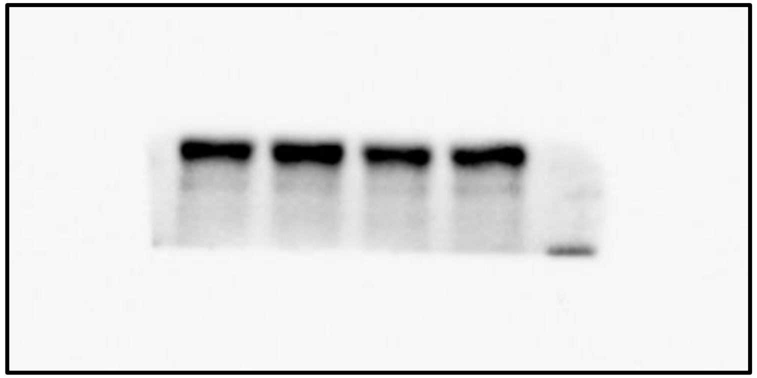

Fig. 5F   n=3

Fig. 5F, SYNCRIP

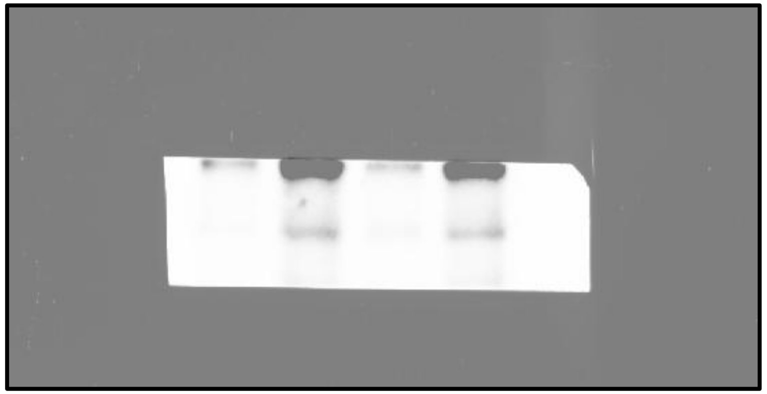

Fig. 5F, DNMT1

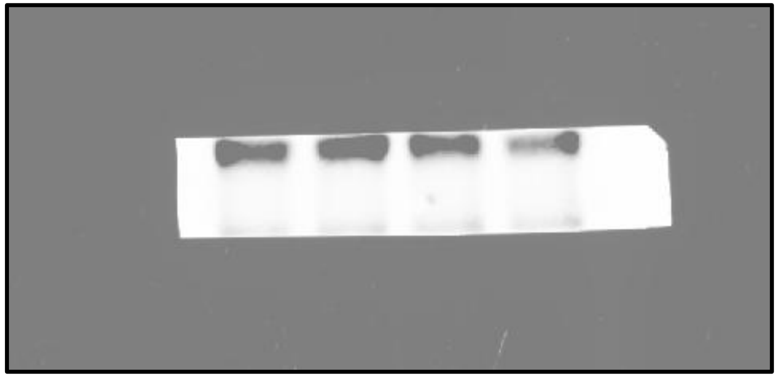

Fig. 5F, DNMT3A

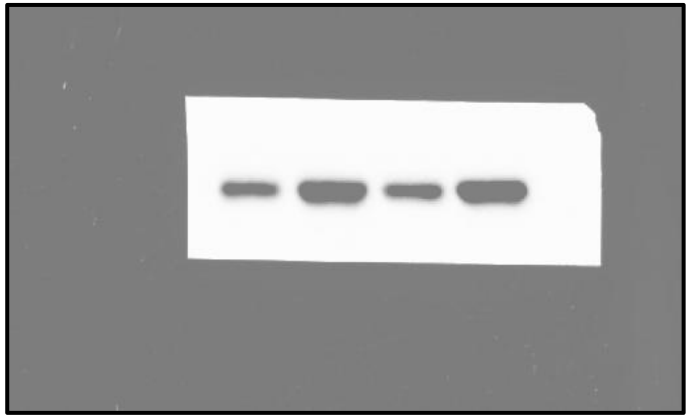

Fig. 5F, DNMT3B

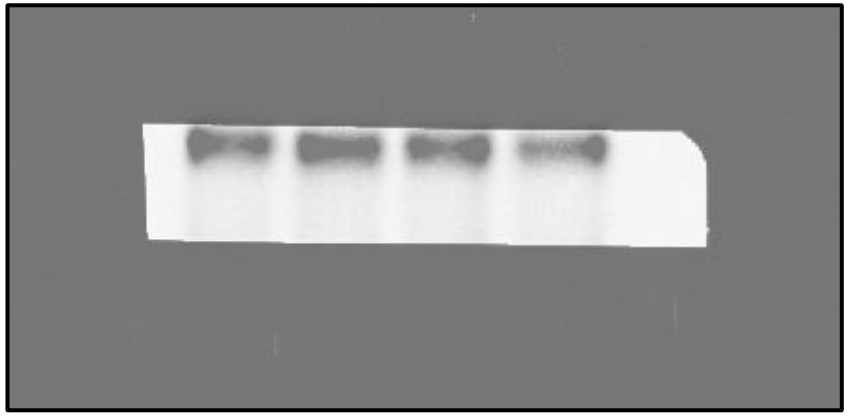

Fig. 5F, GAPDH

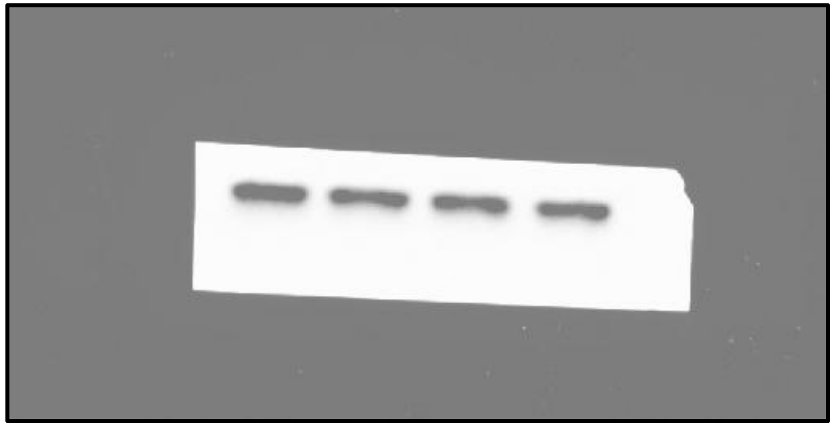

Fig. 6A

Fig. 6A, DNMT3A

Fig. 6A, GAPDH

n=1

n=2

n=3

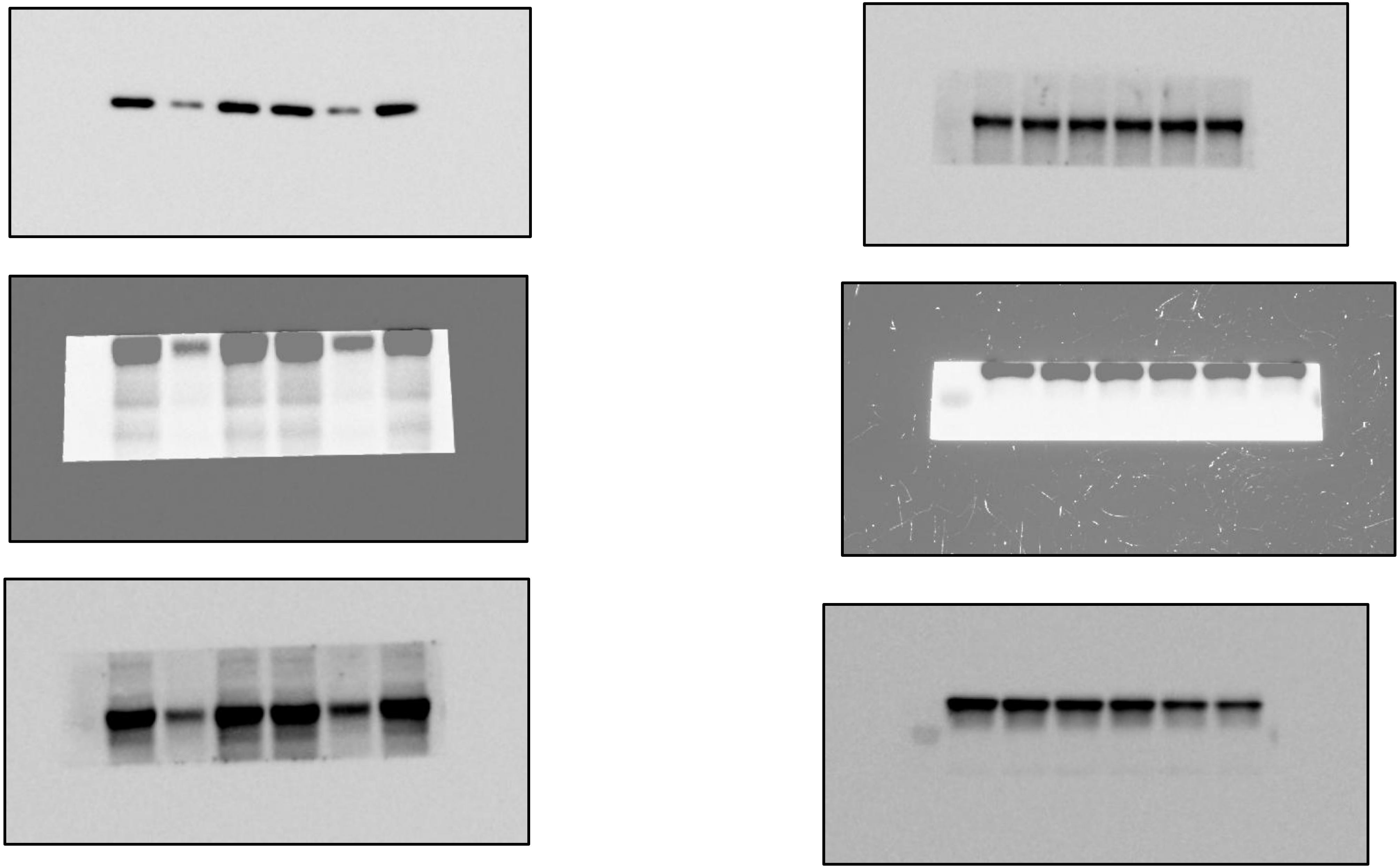

Fig. 7A

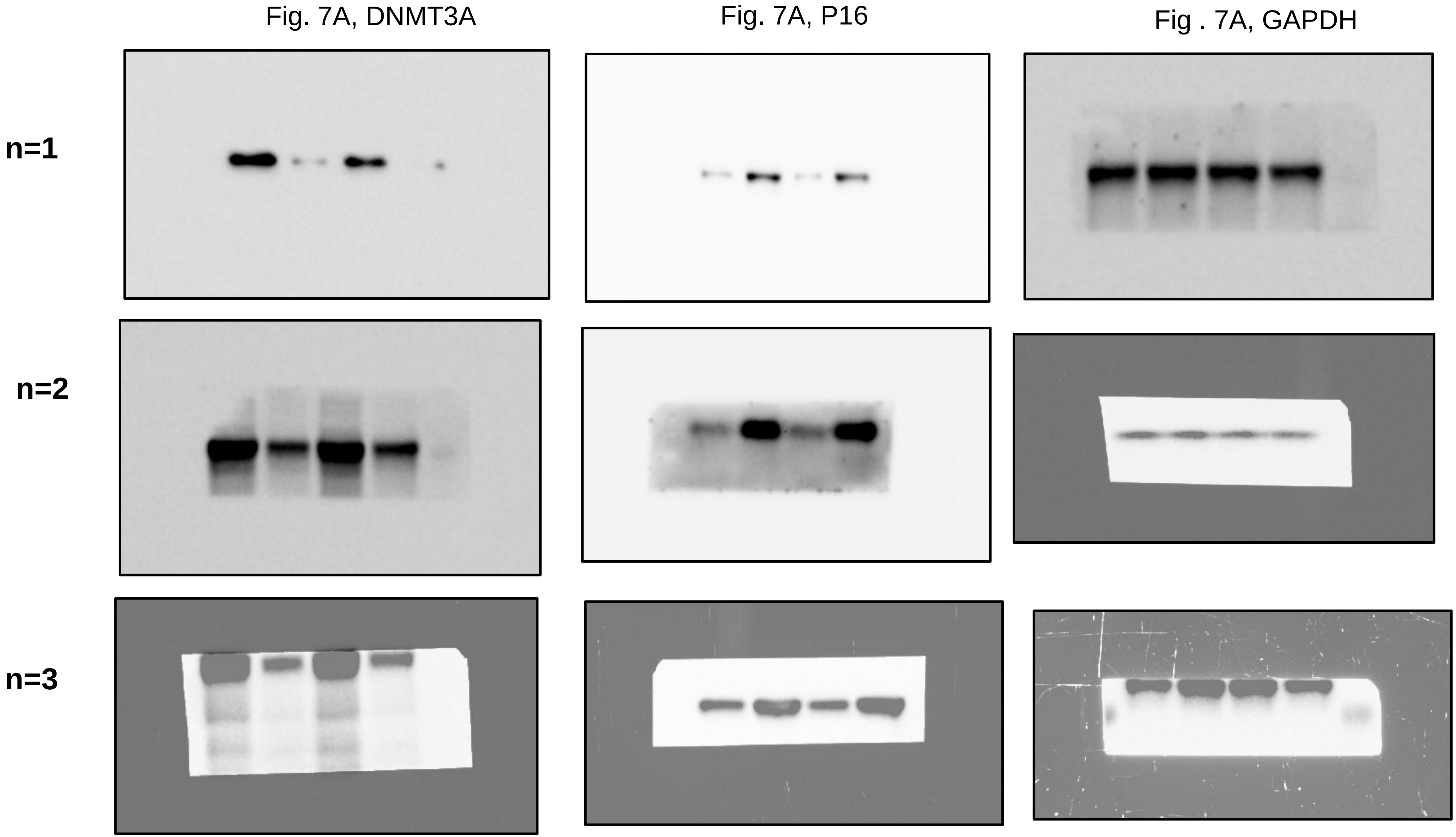

Fig. 7C **n=1**

Fig . 7C, SYNCRIP

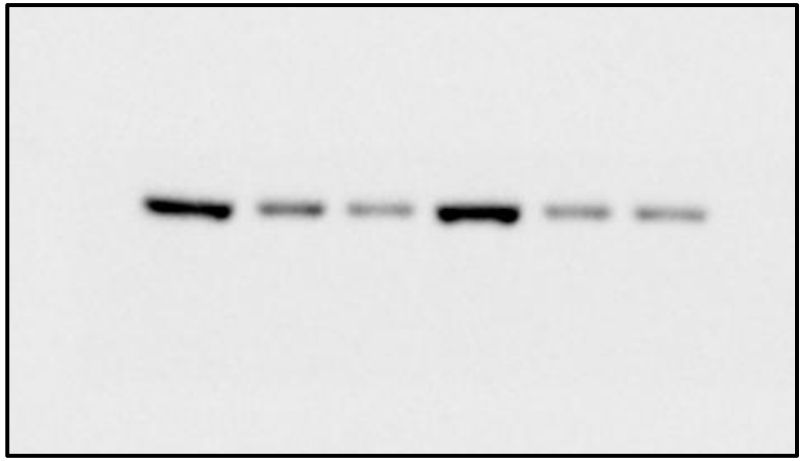

Fig . 7C, DNMT3A

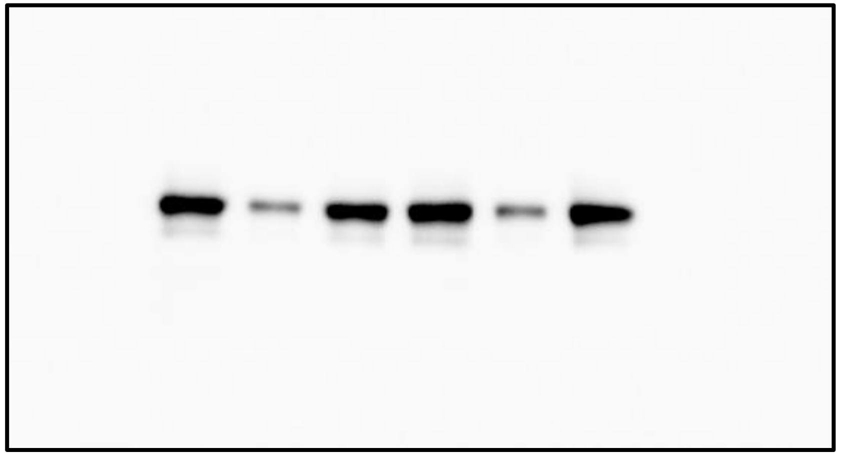

Fig . 7C, P16

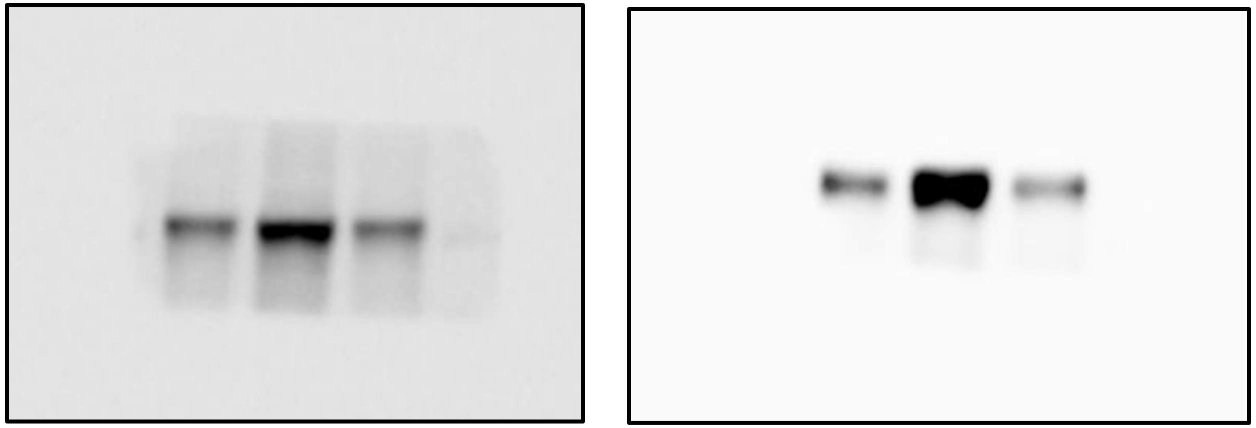

Fig . 7C, GAPDH

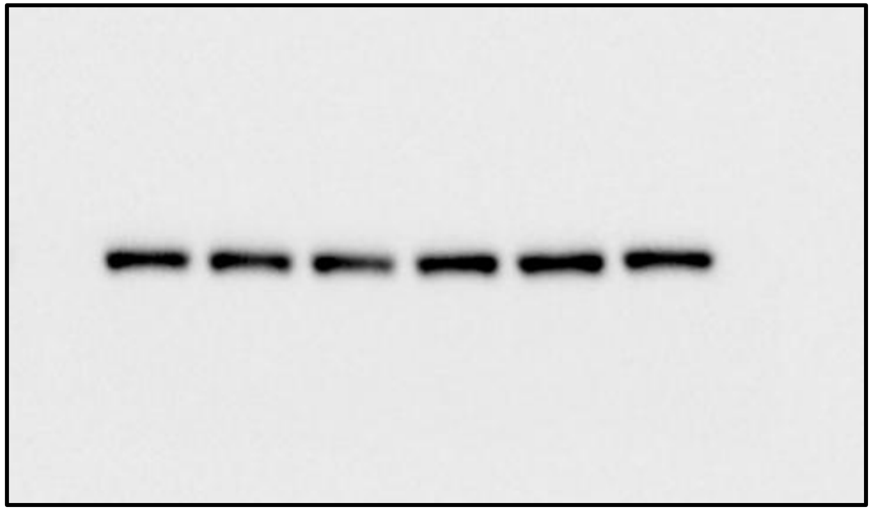

Fig. 7C **n=2**

Fig . 7C, SYNCRIP

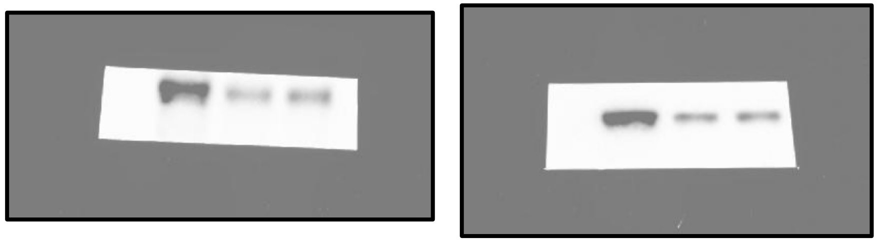

Fig . 7C, DNMT3A

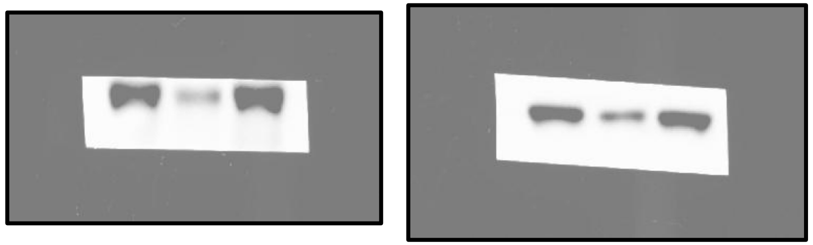

Fig . 7C, P16

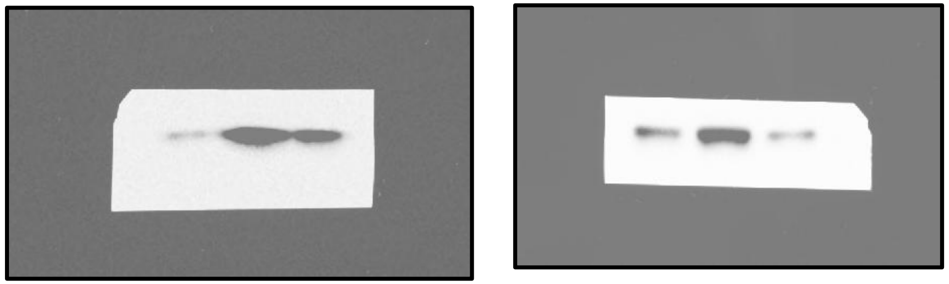

Fig . 7C, GAPDH

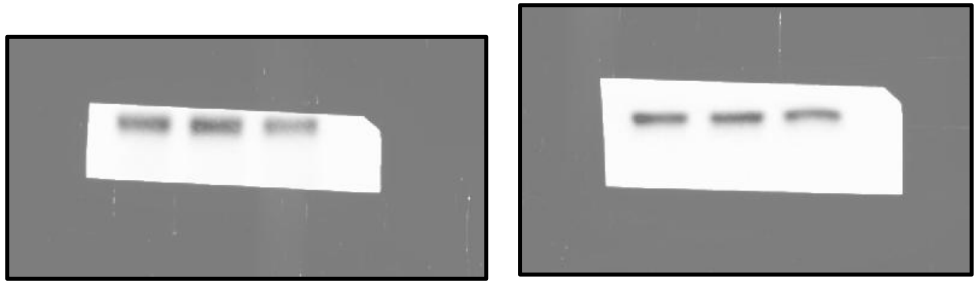

Fig. 7C **n=3**

Fig . 7C, SYNCRIP

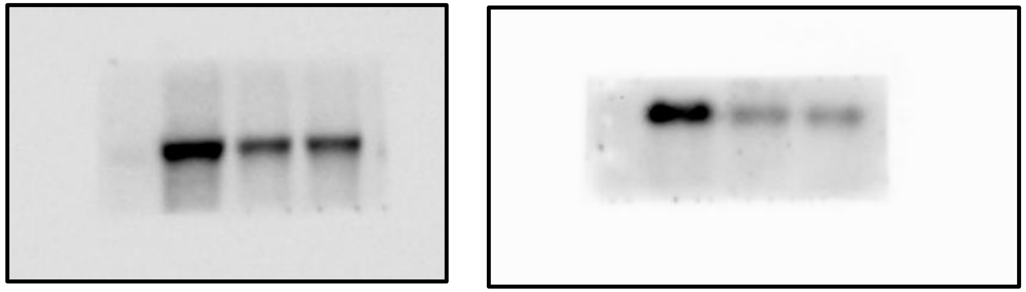

Fig . 7C, DNMT3A

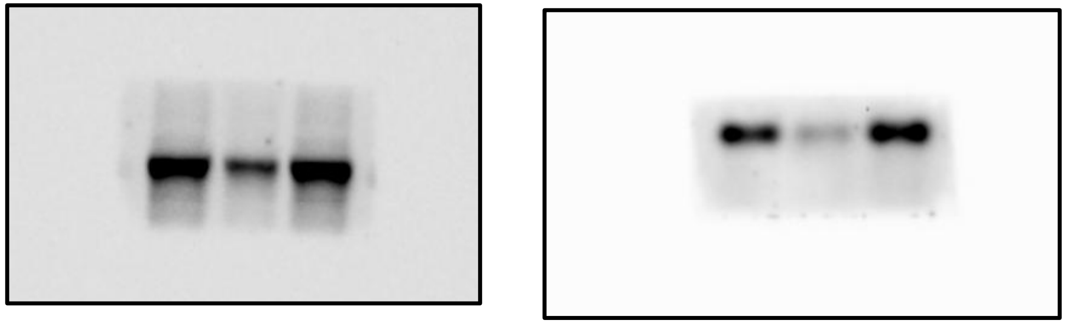

Fig . 7C, P16

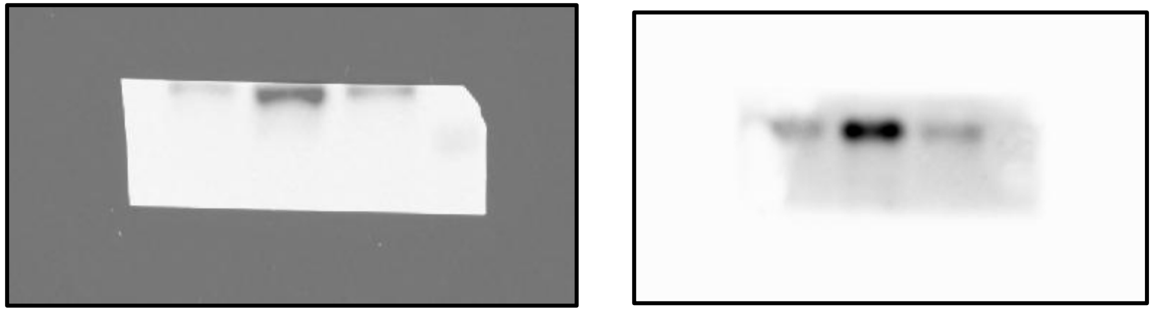

Fig . 7C, GAPDH

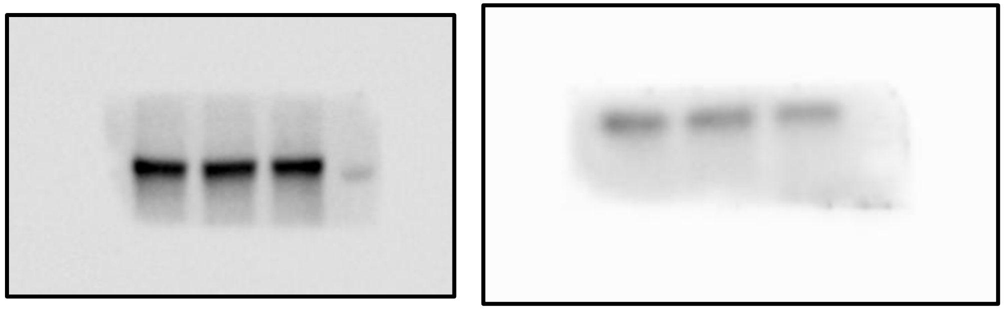

Fig. 8

Fig. 8C, SYNCRIP

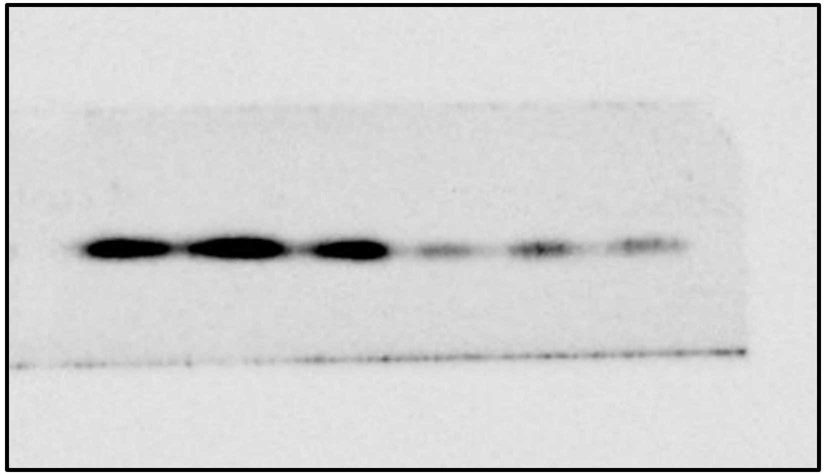

Fig. 8C, DNMT3A

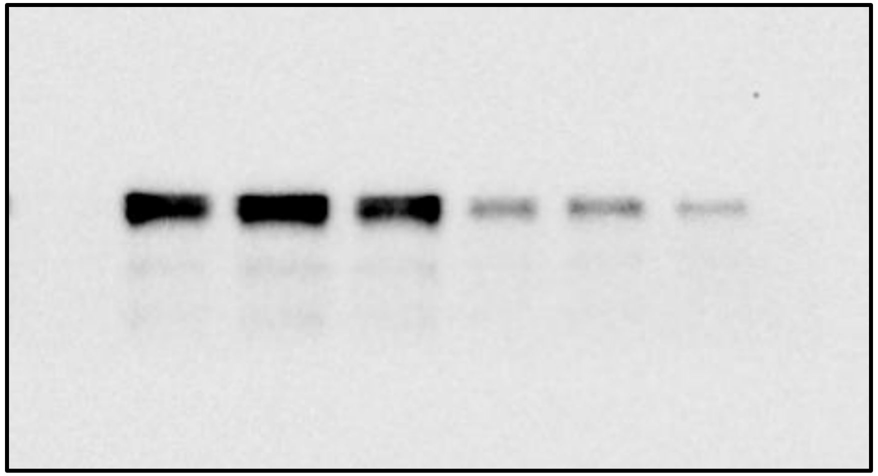

Fig. 8C, P16

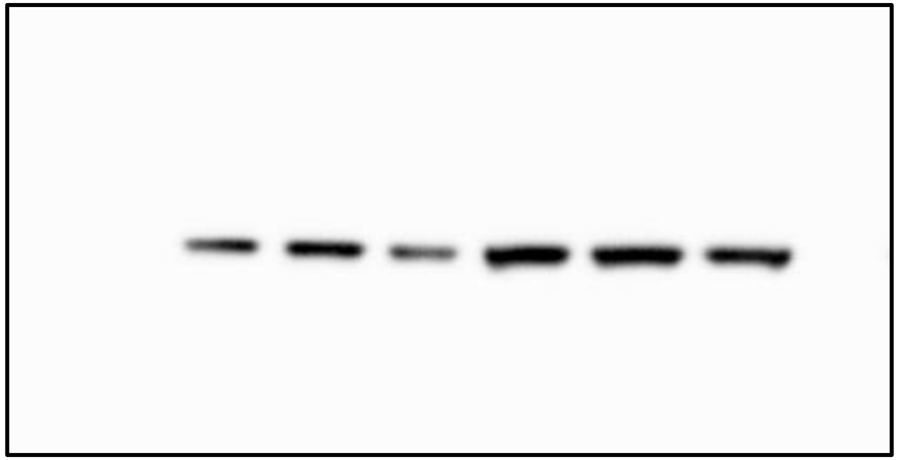

Fig. 8C, GAPDH

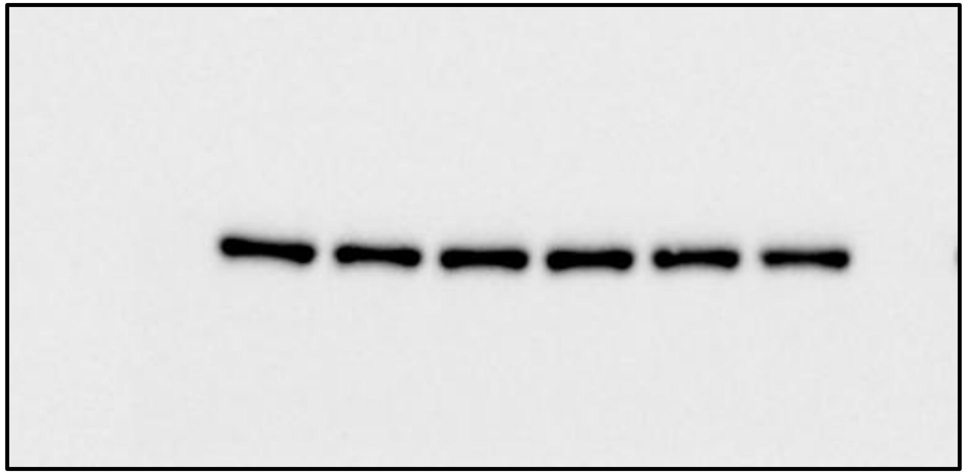

Supplement: Supplementary file 2 — Supplementary Information 1. [file 41598_2024_59575_MOESM2_ESM.pdf]
